# Fiber‐Electrospun Hydrogel Therapy for DNP: A synergistic electrospun‐hydrogel composite for alleviating diabetic neuropathic pain via MMP9 regulation and sodium channel inhibition

**DOI:** 10.1002/btm2.70050

**Published:** 2025-07-28

**Authors:** Wen Chen, Ji Chen, Yingqing Lu, Yangyuxi Chen, Xinxin Liu, Fengrui Yang

**Affiliations:** ^1^ Department of Anesthesiology Yuebei People's Hospital, Shantou University Medical College Shaoguan China; ^2^ Department of Endocrinology Hunan University of Medicine General Hospital Huaihua China; ^3^ Department of Anesthesiology Xiangya Hospital of Central South University Changsha China; ^4^ National Clinical Research Center for Geriatric Disorders, Xiangya Hospital Central South University Changsha China; ^5^ Yali High School International Department Changsha China; ^6^ Department of Anesthesiology Hunan University of Medicine General Hospital Huaihua China

**Keywords:** diabetic neuropathic pain (DNP), electrospinning‐hydrogel composite, Lidocaine (Lid), MMP9 regulation, Sinomenine (SIN), sodium channel inhibition

## Abstract

Diabetic neuropathic pain (DNP) remains a significant challenge in diabetes care, and effective therapeutic strategies are urgently needed. This study introduces an innovative electrospinning‐hydrogel composite, Fiber‐SIN/Gel‐LidC, designed for the controlled and synergistic release of Sinomenine (SIN) and Lidocaine (Lid). Bioinformatics and network pharmacology analyses identified MMP9 as a key player in DNP alleviation. The composite, composed of SIN‐loaded fibers and Lid microcrystals, ensures sustained drug release over 7 days, demonstrating excellent biocompatibility. In vivo experiments on diabetic rats revealed significant improvements in thermal and mechanical pain thresholds, along with a reduction in sciatic nerve excitability. Additionally, the composite significantly attenuated neuroinflammation, neuronal apoptosis, and morphological damage. Mechanistic studies highlighted the neuroprotective effects of Fiber‐SIN/Gel‐LidC, particularly through the regulation of MMP9 and inhibition of sodium channels. These findings suggest that Fiber‐SIN/Gel‐LidC holds great potential as an innovative biomaterial‐based approach for managing DNP, offering promising therapeutic prospects for diabetic neuropathy.

**Translational Impact Statement**

Fiber‐SIN/Gel‐LidC is a novel biomaterial enabling sustained co‐delivery of Sinomenine and Lidocaine to alleviate diabetic neuropathic pain. By targeting MMP9 and sodium channels, it offers a promising therapeutic strategy with strong translational potential.

## 1 INTRODUCTION

Diabetic neuropathic pain (DNP) is a common complication among diabetes patients and significantly diminishes their quality of life.^1^ It is estimated that approximately half of all diabetic patients worldwide experience varying degrees of neuropathy during the course of their illness.^2^ This neuropathy often presents as numbness, pain, or abnormal sensations in the limbs, and can lead to severe functional impairments in advanced cases.^3^, ^4^ The pathogenesis of DNP is complex, primarily involving direct damage to nerve fibers due to persistent hyperglycemia, as well as inflammation and microvascular changes induced by high blood sugar levels.^5^, ^6^, ^7^ These pathological changes affect not only the metabolism and function of the nerve cells themselves but also cause alterations in the surrounding supportive tissues, exacerbating neuropathic pain.^5^

Currently, the treatment of DNP includes pharmacotherapy, physical therapy, and surgical interventions. Commonly used medications such as antidepressants, antiepileptics, and local anesthetics can alleviate symptoms to some extent, but these treatments often come with side effects and limited efficacy.^8^, ^9^, ^10^ Consequently, researchers are continually seeking more effective and safer treatment options.^11^ In recent years, novel drug delivery systems based on nanotechnology and materials science have gained widespread attention for their potential to enhance therapeutic effects and reduce adverse reactions.^12^

Sinomenine (SIN), an alkaloid derived from the traditional Chinese herb Sophora flavescens, has been shown to possess significant anti‐inflammatory and immunomodulatory effects. Its mechanisms likely involve the inhibition of inflammatory mediators and modulation of immune cell activity.^13^, ^14^ Additionally, SIN has demonstrated analgesic effects in models of DNP.^15^ Lidocaine (Lid), a commonly used local anesthetic, reduces neuronal excitability by blocking sodium channels, effectively controlling pain transmission.^16^ This characteristic makes Lid a potential treatment for DNP.^17^ However, single‐agent treatments often fail to achieve long‐term and stable therapeutic effects, thus underscoring the importance of developing a novel drug delivery system that can synergistically release SIN and Lid.^18^

Drug delivery systems developed using nanotechnology and materials science offer new strategies for targeted therapy and controlled drug release.^19^ Particularly, the integration of electrospinning technology with hydrogel materials has enabled the creation of composites with excellent biocompatibility and adjustable release properties.^20^, ^21^ In this study, we utilized electrospinning to produce drug‐loaded fibers with high drug‐loading capacity, which, combined with three‐dimensional hydrogel networks, led to the successful development of a novel Fiber‐SIN/Gel‐LidC composite material. This composite not only effectively stabilizes drug activity and prevents degradation but also exhibits superior controlled‐release characteristics, achieving slow and steady co‐release of drugs, thereby maintaining sustained therapeutic effects.

The primary aim of this research is to develop a new electrospun‐hydrogel composite material (Fiber‐SIN/Gel‐LidC) to achieve synchronized and synergistic release of SIN and Lid, targeting the treatment of DNP. Through in‐depth bioinformatics and network pharmacology analysis, MMP9 was identified as a critical target for both drugs in the treatment of DNP. Building on this discovery, the study further explores how the composite material modulates MMP9 gene expression to treat a rat model of DNP, assessing its effectiveness in alleviating pain and improving neuropathic conditions. We anticipate that this composite material will significantly alleviate neuropathic symptoms in diabetic rats, enhancing their overall quality of life. Moreover, the scientific and clinical significance of this study lies in its innovative approach to DNP treatment, combining advanced biomaterials with precision drug delivery, opening new research avenues for the treatment of neurological diseases and demonstrating broad potential for application and translation.

## 2 MATERIALS AND METHODS

### 2.1 Obtaining transcriptome sequencing data

The gene expression profile dataset GSE95849 was acquired from the Gene Expression Omnibus (GEO) database (http://www.ncbi.nlm.nih.gov/geo/). Differential analysis was performed on peripheral blood samples from six diabetic peripheral neuropathy patients and six healthy control subjects within this dataset. Ethical approval or informed consent was not required, as the data were obtained from a publicly available database.

### 2.2 Search in disease‐related databases

To obtain target genes related to diabetic neuropathic pain (DNP), the Gene Cards database (https://www.genecards.org/) was utilized. The search term “Diabetic neuropathic pain” was entered, with the relevance score set to ≥5 to retrieve highly relevant targets for subsequent analysis.

### 2.3 Candidate target selection

Candidate genes were identified through Venn analysis using the Draw Venn Diagram tool, which compared the candidate targets from the ECTM database, BATMAN‐TCM database, GEO microarray analysis results, and Gene Cards database search results. This analysis yielded potential genes related to Sinomenine (SIN) and Lidocaine (Lid).

### 2.4 Differential gene, protein–protein interaction, and gene ontology combined with Kyoto Encyclopedia of genes and genomes enrichment analysis

Using the limma package in R, differentially expressed genes (DEGs) were identified based on the selection criteria of |log2FC|>1 and *p* < 0.05 using the GEO dataset. All analyses were conducted in R version 4.2.1 (R Foundation for Statistical Computing). A protein–protein interaction (PPI) analysis for target genes and functionally encoded proteins was performed using the String database (https://string-db.org/), and the PPI network was visualized using Cytoscape 3.6.0 software. The co‐expressed genes were subjected to gene ontology (GO) functional enrichment analysis and Kyoto Encyclopedia of genes and genomes (KEGG) pathway enrichment analysis using the clusterProfiler package in R software, with data visualization achieved using the ggplot2 package.

### 2.5 Molecular docking

To evaluate the binding affinity and interaction patterns between candidate drugs/small molecules and their targets, the candidate gene MMP9 was converted into its corresponding protein using the Uniprot database (http://www.uniprot.org/), limited to the species “Human,” and its 3D structure was obtained in pad format. The molecular structures of SIN and Lid were retrieved from the PubChem compound database (https://pubchem.ncbi.nlm.nih.gov/). The target protein receptor was processed by dehydration and the removal of organic substances using PyMOL software. The target protein receptor molecule was prepared using Autodock Vina 1.2.2, which included hydrogenation and charge calculation processes. Both the compound and target protein receptor were converted into “pdbqt” files with appropriate box center and grid parameters. Finally, Vina 1.2.2 was employed to run molecular docking and calculate the docking energy values.

### 2.6 Synthesis and characterization of LidC

Lid (100 mg) (BP214, CAS: 6108‐05‐0, Sigma‐Aldrich) was dissolved in acetone (10 mL) (AX0120T, CAS: 67‐64‐1, Sigma‐Aldrich) overnight, with pre‐sonication for 10 min. The LidC‐acetone solution was rapidly poured into a pre‐cooled polyvinyl alcohol (PVA) solution (0.5% w/v, PVA solution: LidC‐acetone solution volume ratio = 10:1) (360627, CAS: 9002‐89‐5, Sigma‐Aldrich) and stirred for 2 h in an ice bath. The resulting precipitate obtained after centrifugation at 10,000*g* for 20 min was freeze‐dried, yielding LidC microcrystals with a length of approximately 20 μm. The size of the microcrystals was adjusted by varying the stirring time. The morphology of LidC was characterized using scanning electron microscopy (S‐4800, Hitachi, acquired from Shanghai Fluorescent Optical Technology Co., Ltd.). The crystal structure of LidC was determined by X‐ray diffraction (XRD, PANalytical, XPert Pro), and the interaction between functional groups in the sample was recorded using an FTIR spectrometer (912A0770, ThermoFisher, USA). The drug loading of LidC was determined using a UV‐visible spectrophotometer (Shimadzu, UV‐2600).

### 2.7 Preparation and characterization of PLGA‐SIN electrospun fibers

Poly (lactic‐co‐glycolic acid) (PLGA) (719927, CAS: 26780‐50‐7, Sigma‐Aldrich) and SIN (C₁₉H₂₃NO₄, CAS: 115‐53‐7; purity >99%, purchased from BioBioPha) were dissolved in different ratios in a DCM/DMF 70/30 solution (dichloromethane/N, N‐dimethylformamide = 70/30) or a DCM/MeOH 70/30 solution (dichloromethane/methanol = 70/30). The solutions were stirred at room temperature for 2 h using a magnetic stirring bar (MYP84‐1, Shanghai, China). PLGA‐SIN electrospun fibers were prepared by adjusting the process parameters such as needle type, injection solution flow rate, electrostatic field intensity, collector rotation speed, and distance between the needle and the rotating collector. The drug loading capacity (DLC) and drug loading efficiency (DLE) of SIN were determined by dissolving and drying the Fiber‐SIN in methanol and extracting the absorbance at 343 nm from the obtained absorption spectra. The DLC and DLE of SIN were calculated using the formulas: DLC (%) = (mass of loaded drug)/(total mass of fibers) × 100%, DLE (%) = (mass of loaded drug)/(initial mass of drug) × 100%.

### 2.8 Preparation of F127 hydrogel

A series of different concentrations (20%, 25%, 30%, 35%, and 40% wt%) of F127 solution was prepared by dissolving Pluronic® F127 (P2443, CAS: 9003‐11‐6, Sigma‐Aldrich) in sterile water. The solution was manually stirred for 20 min to completely dissolve F127, followed by freezing at −80°C for 5 min and storing in a refrigerator at 4°C. A 40% F127 solution was chosen to produce thermosensitive injectable hydrogel.

### 2.9 Fabrication of Fiber‐SIN/Gel‐LidC composite material

To prepare the Fiber‐SIN and LidC composite material, Fiber‐SIN and LidC were added to a 40% wt% F127 solution at 4°C, and the solution was vortexed to ensure thorough mixing. The mass ratio of SIN to LidC in the composite material was adjusted within certain proportions. The suspension was vortexed for 1 h at 4°C to obtain a homogeneous mixture. Subsequently, the suspension was maintained in a constant temperature water bath at 37°C for 10 min to obtain the f‐SIN/g‐LidC composite material.

### 2.10 Characterization of Fiber‐SIN/Gel‐LidC composite material

The surface morphology of freeze‐dried PLGA fibers (loaded or unloaded with SIN), F127 hydrogel, and Fiber‐SIN/Gel‐LidC composite material was examined using a scanning electron microscope (SEM; S‐4800, Hitachi, purchased from Shanghai Fulai Optical Technology Co., Ltd.). The accelerating voltage was set at 20 kV.

Furthermore, FITC (F7250, CAS: 3326‐32‐7, Sigma‐Aldrich) was used to label Fiber‐SIN and Gel‐LidC, and the distribution of labeled fibers in the Fiber/Gel composite material and F127 hydrogel was characterized using a fluorescence microscope (Ni‐U, Nikon Corporation, Japan). Briefly, for the preparation of FITC‐labeled fibers (Fiber‐SIN‐FITC), FITC (1% by weight) was added to a solution containing PLGA (20% by weight) and SIN (60% by weight) and electrospun to generate fibers. RhB (Gel‐LidC‐RhB) was labeled into F127 hydrogel by uniformly mixing RhB (1% by weight) with F127‐LidC solution at 4°C. The Fiber‐SIN‐FITC/Gel‐LidC‐RhB composite material was then obtained using the aforementioned method. Finally, the rheological properties, storage modulus (G′), and loss modulus (G″) of the Fiber‐SIN/Gel‐LidC composite material were measured using a HAAKE MARS 60 dynamic shear rheometer (ThermoScientific, USA) at a strain of 1%, frequency of 1.0 Hz, and a heating rate of 2°C/min.

### 2.11 In vitro drug release of Fiber‐SIN/Gel‐LidC composite material

The drug release test lasted for 14 days. A transfer of 1 mL of Fiber‐SIN/Gel or Fiber/Gel‐LidC composite solution was made to a dialysis bag (MWCO, 3500 Da, Sigma‐Aldrich, USA), which was then placed inside the dialysis bag. Gelation was achieved by allowing the solution to stand for 10 min at 37°C. Phosphate‐buffered saline (PBS) solution, with or without 20 U/mL lipase (L3170, Sigma‐Aldrich, USA), was slowly added to two sets of vials (*n* = 3 per group) containing the composite to be dialyzed. Subsequently, the vials were incubated at 37°C on a shaker incubator at a speed of 40 rpm. At specific time points, 300 μL of dialysate was withdrawn and replaced with 300 μL of fresh PBS solution. Concentrations of Lid and SIN released into buffer solutions were determined using a UV–visible spectrophotometer at wavelengths of 263 and 343 nm, respectively. Standard solutions of Lid and SIN were used to generate linear calibration curves, which were then employed to quantify the concentrations of Lid and SIN in all test samples.

#### 2.11.1 In vitro degradation experiment of composite material

Photographs were taken with a digital camera, and the weight method was used to evaluate the in vitro degradation of Fiber/Gel. After 24 h of lyophilization, the initial morphology of Fiber was recorded, and an equivalent weight (W0) was determined. Subsequently, 15 mL of PBS solution (with or without 20 U/mL lipase) was added to vials containing Fiber/Lid and incubated at 37°C on a shaker at a speed of 40 rpm. The solution in the vials was changed every 3 days. At specific time points, the remaining weight (W1) of the composite material was recorded. The formula for calculating the percentage of remaining weight is as follows: Remaining weight (%) = W1/W0 × 100%. Additionally, to investigate the in vitro diffusion of F127 gel, 1% RhB was added to the gel for visual observation. The morphology of Gel‐RhB was recorded with a digital camera at predetermined time points.

#### 2.11.2 Biocompatibility and pharmacokinetic study of the composite material in vivo

After the completion of four treatment cycles, a blood routine examination was conducted using the Sysmex XT‐1800i hematology analyzer (Sysmex Corporation, Japan). The levels of aspartate aminotransferase, alanine aminotransferase, alkaline phosphatase, gamma‐glutamyl transpeptidase, blood urea nitrogen, and creatinine in the serum were measured using the FUJIFILM DRI‐Chem 7000 clinical chemistry analyzer (Fujifilm Corporation, Japan). Additionally, hematoxylin–eosin (H&E) staining was performed to monitor the histological changes in major organs. To evaluate the in vivo release kinetics of the Fiber‐SIN/Gel‐LidC system, the serum concentrations of SIN and Lid in diabetic rats were measured by LC–MS/MS. Blood samples were collected at 0.5, 1, 2, 4, 8, 12, 24, 48, 72, 120, and 168 h post‐administration. Samples were processed using acetonitrile precipitation, and SIN and Lid concentrations were quantified by liquid chromatography–tandem mass spectrometry (LC–MS/MS). Pharmacokinetic parameters such as C_max, T_max, and t_1/2 were calculated accordingly.

### 2.12 Cell experimentation and grouping

RSC96 cells (CRL‐2765, ATCC) were used to investigate the impact of Fiber‐SIN/Gel‐LidC on neuronal cells in vitro. The cells were cultured in low‐glucose Dulbecco's Modified Eagle Medium (DMEM‐LG, 10567014, ThermoFisher) supplemented with 100 U/mL penicillin–streptomycin (15140148, ThermoFisher) and 10% fetal bovine serum (10100147C, ThermoFisher) at 37°C, 5% CO_2_. Passage 3 RSC96 cells were harvested using 0.25% trypsin, centrifuged at 800 rpm for 5 min, resuspended in DMEM‐LG medium at a density of 3 × 10^6^ cells/mL, and then seeded onto 100 mm culture dishes. After 24 h of incubation, the RSC96 cells were cultured in different media. The basic experiment consisted of three groups: (1) Control group: RSC96 cells cultured in normal medium, (2) HG group: RSC96 cells cultured in medium containing 50 mM high glucose (11965118, ThermoFisher), and (3) HG + Fiber‐SIN/Gel‐LidC group: RSC96 cells cultured in medium containing 50 mM high glucose and 10 mg Fiber‐SIN/Gel‐LidC. The cells were incubated at 37°C for 48 h under different treatments.^22^

### 2.13 Development of a rat model for DNP

Given that female rats demonstrate longer durations of pain behavior compared to males,^23^ we selected 30 female Sprague–Dawley (SD) rats aged 7–8 weeks and weighing 180 ± 20 g for the experiments. Prior to the experiments, all animals underwent a 7‐day acclimatization period under standard laboratory conditions. Randomization into control and model groups was performed using a random number generator to minimize selection bias. Additional randomization was conducted at the start of the experiment to further ensure allocation concealment. Throughout data collection and analysis, experimenters remained blinded to group assignments to reduce potential bias. All animals were housed in a barrier facility under a 12‐h light/dark cycle, at 25 ± 2°C and 60%–70% relative humidity, with free access to food and water. All rats were housed in a controlled environment with a temperature of 25 ± 2°C, relative humidity of 60%–70%, and 12 h cycles of light and dark. They had free access to food and water. After a 7‐day adaptation period, the rats were randomly assigned into control and model groups. This study was approved by the Animal Ethics Committee of Hunan University of Medicine General Hospital (Approval No. 202403055) and complied with national and international guidelines on the ethical use of laboratory animals.

To construct a rat model for DNP, we injected streptozotocin (Sigma‐Aldrich, Shanghai, China) into the rat's abdominal cavity at a dose of 60 mg/kg. Rats in the control group received an injection of saline. After 72 h of streptozotocin administration, we measured the rats' fasting blood glucose and insulin levels. Then, we selected rats with fasting blood glucose levels within the range of 300–350 mg/dL for further study. We measured their body weight, blood glucose and insulin levels, as well as behavioral parameters (thermal hyperalgesia and mechanical allodynia). Rats that met the criteria for DNP were included in the DNP group.

Experimental groups were as follows:

**Control group**: Rats were fed normally and received an intra‐abdominal injection of PBS as a control for the DNP group's STZ injection.

**DNP group**: Rats were injected with STZ to induce the model. After 4 weeks, rats with fasting blood glucose levels between 300 and 350 mg/dL and obvious signs of pain behavior were included in the model group.

**Control + PBS group**: Rats from the control group received a PBS injection at the sciatic nerve site, 0.5 mL per injection, once every 7 days for a total of 4 cycles, serving as a control for the DNP + PBS group.

**DNP + PBS group**: Rats from the DNP group received a PBS injection at the sciatic nerve site, 0.5 mL per injection, once every 7 days for a total of 4 cycles, serving as a control for the DNP + f‐SIN/g‐LidC group.

**DNP + f‐SIN/g‐LidC group**: Rats from the DNP group were injected with STZ to induce the model. After 4 weeks, rats with fasting blood glucose levels between 300 and 350 mg/dL and obvious signs of pain behavior received an injection of f‐SIN/g‐LidC at the sciatic nerve site, 0.5 mL per injection, once every 7 days for a total of 4 cycles.

At the end of the study, animals were anesthetized and euthanized with pentobarbital sodium (50 mg/kg, C8383, Sigma‐Aldrich, USA). Small dorsal root ganglia (DRG) from the sciatic nerve and surrounding muscle tissue were harvested and stored at −80°C for future use.^24^

### 2.14 Estimation of body weight, blood glucose, and serum insulin levels

At the beginning of the study, the weight of each animal was measured, and animals with similar weights were grouped. Randomization of animals was conducted at the initiation of the treatment regimen. Regular weight measurements were taken for each group until the study concluded. Fasting blood glucose levels were assessed every 3 days using a commercial blood glucose kit (S0201S, Beyotime). Plasma insulin levels in anticoagulated blood vessels were determined using an insulin enzyme‐linked immunosorbent assay kit (ERINS, ThermoFisher, USA).

### 2.15 Immunofluorescence

Cells or tissues were washed with ice‐cold PBS and fixed with 4% paraformaldehyde (P885233, Macklin) for 15–30 min. Subsequently, 0.1% Triton (L885651, Macklin, USA) was applied for 15 min. After two washes with PBS, cells or tissues were incubated overnight at 5°C in PBS containing 15% FBS. The following antibodies and dyes were then added and incubated overnight at 4°C, covering the cells or tissues: mouse anti‐CD3 (1:100, 14‐0030‐82, ThermoFisher, USA), mouse anti‐MMP9 (1:100, MA5‐45511, ThermoFisher, USA), mouse anti‐Iba‐1 (1:100, ab283319, Abcam), mouse anti‐TRPV1 (1:100, ab203103, Abcam), mouse anti‐c‐Fos (1:1000, ab208942, Abcam), mouse anti‐NF‐H (2 μg/mL, 711025, ThermoFisher, USA), rabbit anti‐MPO (1:100, ab208670, Abcam), rabbit anti‐Arg‐1 (1:500, ab96183, Abcam), rabbit anti‐NeuN (1:500, ab177487, Abcam), rabbit anti‐iNOS (1:500, ab178945, Abcam), and rabbit anti‐MBP (1:50, PA1‐10008, ThermoFisher, USA). After three washes with TBST (TBS containing 1% Tween‐20), cells were incubated with goat anti‐mouse Alexa Fluor™ 488 secondary antibody (A‐11001, ThermoFisher, USA) and goat anti‐rabbit Alexa Fluor™ 555 secondary antibody (A‐21428, ThermoFisher, USA). Subsequently, cells were stained with DAPI and observed under a fluorescence microscope (Zeiss Observer Z1, Germany). Target areas were selected for fluorescence intensity measurements in the images, which were further processed and quantified using ImageJ to determine the number of positive cells.

### 2.16 Enzyme‐linked immunosorbent assay

Quantification of inflammatory cytokines in neural tissue was performed using the IL‐1β ELISA kit (ab255730, Abcam, UK), IL‐6 ELISA kit (ab234570, Abcam, UK), and IL‐10 ELISA kit (ab214566, Abcam, UK). Initially, the antigen was diluted to an appropriate concentration in the coating diluent, and 5% bovine serum (F8318, MSK, Wuhan, China) was incubated at 37°C to block the enzyme reaction wells for 40 min. Subsequently, diluted samples were added to the enzyme reaction wells, followed by the addition of enzyme‐labeled antibodies and substrate solution. Finally, 50 μL of stop solution was added to each well, and the experimental results were measured within 20 min. The plate was read at 450 nm using a plate reader (Bio‐Rad, USA), and the quantification of inflammatory cytokines was performed according to the manufacturer's instructions using the respective assay kits. The measured optical density values were converted to concentration values, and a standard curve was plotted for data analysis.

### 2.17 Histological staining methods

#### 2.17.1 Nissl staining

Neurological tissue samples were fixed in a 4% formalin solution for 48 h. After fixation, the samples were sequentially immersed in 70%, 80%, and 90% ethanol solutions for 30 min each, followed by two immersions in 95% and 100% ethanol solutions for 20 min each. The dehydrated samples were then infiltrated with xylene, embedded in paraffin, and cut into 5 μm sections, which were subsequently deparaffinized. Nissl staining solution (C0117, Beyotime, China) was used to stain the sections, which were then analyzed under a light microscope to capture images of neurons with visible nuclei and relatively intact cell morphology.

#### 2.17.2 TUNEL staining

TUNEL staining was performed to evaluate cell apoptosis in neurons using a one‐step TUNEL cell apoptosis detection kit (11684795910, Roche, USA), following the manufacturer's instructions. Slide mounts were briefly incubated with 0.5% Triton X‐100 at room temperature for 10 min to facilitate tissue permeabilization. They were then incubated with the TUNEL reagent mixture at 37°C for 1 h. Afterward, the slide mounts were counterstained with DAPI for 10 min, coverslipped, and observed under a fluorescence microscope (IX73, Olympus, Japan). ImageJ software was used for image processing and quantitative analysis of positive areas.

#### 2.17.3 H&E staining

Neurological tissue samples were fixed and sectioned, and the wax was removed by dipping the wax blocks into xylene. Dehydration was performed using 100%, 95%, and 70% ethanol, followed by either mounting or water rinsing. The prepared sections were stained with H&E staining solution (H8070, Solarbio, Beijing, China) for 5–10 min at room temperature. Subsequently, the sections were rinsed with distilled water, dehydrated in 95% ethanol, and placed in an eosin staining solution (G1100, Solarbio, Beijing, China) for 5–10 min. After routine dehydration and coverslipping, images were captured using a Zeiss Axioscan slide scanner (Carl Zeiss, Oberkochen, Germany).

#### 2.17.4 Toluidine blue staining

Neurological tissue samples were fixed in 10% formaldehyde solution for 1 h. After fixation, the samples were washed with phosphate‐buffered saline to remove residual formaldehyde. The fixed samples were then soaked in a 0.1% toluidine blue solution for 30 min. After removal, the samples were washed with phosphate‐buffered saline multiple times to remove excess toluidine blue dye. The stained samples were placed on microscope slides, a drop of mounting medium was added, and a coverslip was applied. Abnormal myelinated axons, characterized by demyelination, axon atrophy, disorganized myelin sheath, and vacuolation, were manually counted and expressed as a percentage (abnormal axons/total axons).

### 2.18 Observation of neural microstructure using TEM

The supermicrostructure of the myelin sheath was observed and evaluated using a transmission electron microscope (TEM, FEI, USA). Morphometric measurements were performed on TEM images using ImageJ software. The morphological parameters assessed included axon diameter, myelin sheath diameter, and Gratio (axon diameter/myelin sheath diameter). These parameters provide an accurate and objective evaluation of myelin sheath and axonal damage.

### 2.19 Immunohistochemistry

Rat neural tissue sections were obtained for analysis and underwent fixation and embedding. The embedded tissues were then sliced, followed by the dewaxing process to remove the wax and make the sections hydrophilic, facilitating subsequent immunostaining procedures. Dewaxed tissue sections were treated with specific His48 antibody (14‐0570‐82, 0.5 mg/mL; ThermoFisher, USA), followed by treatment with Anti‐Rabbit‐HRP secondary antibody (12‐348, 1:1000; Sigma Aldrich, USA). The locations where the secondary antibody bound to the first antibody were visualized using a DAB staining reagent (ab64238, Abcam, USA). After staining, the tissue sections were dewaxed and mounted for observation. Six animals were used in each group, with one section stained per animal and one field of view chosen for imaging. Following imaging, ImageJ 1.48u software (V1.48, National Institutes of Health, USA) was used to calculate the percentage of positive area.

### 2.20 Detection of protein expression levels using Western blot

Total protein was extracted from rat neural tissue. First, cultured cells from each group were digested using trypsin (Sigma‐Aldrich, Shanghai, China, T4799‐5G) and collected. Next, an enhanced RIPA lysis buffer containing a proteinase inhibitor (Dr. De Company, Wuhan, China, AR0108) was used for cell lysis. The protein concentration was measured using the BCA protein quantification kit (Dr. De Company, Wuhan, China, AR1189). Proteins were separated by SDS‐PAGE and transferred onto a Polyvinylidene Fluoride (PVDF) membrane (Sigma Aldrich, ISEQ07850). The membrane was blocked with 5% BSA (Solarbio, Beijing, China, 9048‐46‐8) for 1 h at room temperature. The following primary antibodies, diluted as specified, were added: Cc3 (Cleaved Caspase‐3) (ThermoFisher, 1:500, PA5‐96077), Bcl2 (ThermoFisher, 1:1000, PA5‐27094), Bax (ThermoFisher, 1:100, MA5‐14003), MMP9 (Abcam, UK, 1:1000, ab76003), COX2 (ThermoFisher, 1:1000, PA5‐86956), Iba‐1 (Abcam, UK, 1:1000, ab178846), Arg‐1 (Abcam, UK, 1:1000, ab203490), iNOS (Abcam, UK, 1:1000, ab178945), TRPV1 (Abcam, UK, 1:1000, ab305299), Nav1.7 (Abcam, UK, 1:1000, ab272931), Nav1.8 (Abcam, UK, 1:4000, ab63331), Kv1.1 (Abcam, UK, 1:1000, ab252537), Kv2.1 (Abcam, UK, 1:1000, ab192761), Cav1.3 (Abcam, UK, 0.5 μg/mL, ab191038), and GAPDH (Sigma Aldrich, Shanghai, China, 1/2000, SAB2108668). The membrane was then washed three times with PBST for 5 min each wash. Subsequently, the Anti‐Mouse‐HRP secondary antibody (CST, USA, Cat # 7076, 1/5000) or Anti‐Rabbit‐HRP secondary antibody (CST, USA, Cat # 7074, 1/5000) was added and incubated at room temperature for 1 h. The membrane was washed again three times with PBST for 5 min each wash. PBST was discarded, and an appropriate amount of ECL working solution (Omt‐01, Beijing Oumijia De Medical Technology Co., Ltd., Beijing, China) was added. The membrane was incubated at room temperature for 1 min, and excess ECL reagent was removed. The membrane was covered with plastic wrap, sealed, and placed in a dark box. After 5–10 min of exposure, the membrane was developed and fixed. Finally, the ChemiDoc Touch imaging system was used to capture the blot images, and ImageJ software was used for quantitative analysis of the bands.

### 2.21 Assessment of mechanical stimulus‐induced pain

The assessment of mechanical stimulus‐induced pain was conducted in a ventilated polycarbonate chamber. Rats were placed on an elevated aluminum mesh platform and allowed to acclimate to the apparatus for 60 min prior to testing. Mechanical allodynia was evaluated using von Frey filaments (North Coast Medical, Morgan Hill, CA) employing an “up‐and‐down” method. The plantar surfaces of each hind paw were stimulated five times with five different weights of filaments (0.16–4.0 g). The stimulation started with a 0.6 g filament, gradually increasing until the rat responded by licking and/or lifting its paw off the testing surface. If the rat responded positively to three or more stimuli, it was coded as a positive response. Once a positive response was detected, lighter filaments were used to assess the sensory threshold of each paw.

### 2.22 Assessment of thermal stimulus‐induced pain

Rats were placed on a hot plate analgesia tester (BME‐480, Shanghai Yuyan, China) with a temperature set at 50 ± 0.5°C, and a transparent barrier was placed to prevent rat escape. The time when rats displayed licking, paw withdrawal, or jumping in response to the thermal stimulus was recorded as the paw withdrawal thermal latency (PWTL). To avoid hypersensitivity and skin damage, a cut‐off time of 30 s was set. When the time reached 30 s, the rat was removed from the hot plate and the PWTL was recorded as the maximum value, that is, 30 s. Each thermal stimulus was repeated three times with a 5‐min interval between stimuli, and the average PWTL was calculated. The percentage of maximum possible effect (%MPE) of sensory blockade was calculated as: %MPE = (PWTL1 − PWTL0)/(cut‐off time − PWTL0) × 100%. Here, PWTL1 refers to the PWTL after injection, PWTL0 refers to the baseline PWTL, and the cut‐off time is 30 s. When %MPE reached 50%, it indicated effective sensory blockade, and the duration of sensory blockade was measured from the time %MPE reached 50% to the time it dropped below 50%.

### 2.23 Complete DRG preparation and electrophysiological recording

To prepare the DRG (Dorsal Root Ganglion), rats were anesthetized with 1% pentobarbital sodium (50 mg/kg body weight, intraperitoneal injection). The lumbar DRGs were carefully dissected from the sciatic nerve and placed in artificial cerebrospinal fluid (ACSF) maintained at 4°C (see composition below). After removal of connective tissue, the ganglia were digested with a mixture of 0.4 mg/mL pancreatin (15400054, ThermoFisher) and 1.0 mg/mL type A collagenase (SCR136, Sigma‐Aldrich) at 37°C for 40 min, while continuously oxygenated (O_2_) with 95% and 5% CO_2_ gentle bubbling. Following digestion, the ganglia were transferred to ACSF and incubated at 28°C under a mixed gas atmosphere.

The intact DRGs were incubated in ACSF for at least 1 h and then transferred to the recording chamber. During recording, the ganglia were anchored in place with a small anchor and submerged in a chamber filled with an external solution saturated with mixed gas. The temperature was maintained at 26–28°C. Dorsal root ganglion neurons were visualized using a 40× water immersion objective attached to a BX51WI microscope (Olympus, Tokyo, Japan) equipped with an infrared differential interference contrast optical device. Whole‐cell recordings were performed using an Axon 200B amplifier (Molecular Devices, USA). Patch pipettes (4–7 MΩ) were pulled from borosilicate glass capillaries using a two‐step vertical puller (model PP‐83, Narishige, Tokyo, Japan), resulting in a series resistance of approximately 10 MΩ. The pipette offset was adjusted using the 200B Commander software (Molecular Devices) to online correct for junction potentials. Voltage errors were minimized by 80%–90% series resistance compensation, and capacitive transients were eliminated using a membrane patch clamp amplifier. Offline linear leak subtraction was performed. Neurons with resting membrane potentials more negative than −50 mV and displaying overshooting action potentials (APs) were selected for further study.

The ACSF composition (in mM) was as follows: 124 NaCl (S1679, Sigma‐Aldrich), 2.5 KCl (12636 Sigma‐Aldrich), 1.2 NaH_2_PO_4_ (S0751, Sigma‐Aldrich), 1.0 MgCl_2_ (M4880, Sigma‐Aldrich), 2.0 CaCl_2_ (C5670, Sigma‐Aldrich), 25 NaHCO_3_ (S5761, Sigma‐Aldrich), and 10 glucose (49163, Sigma‐Aldrich). The internal pipette solution used for current clamp recording and potassium current measurements contained (in mM): 140 KCl, 2 MgCl_2_, 10 HEPES (H4034, Sigma‐Aldrich), 2 Mg‐ATP (A6419, Sigma‐Aldrich) (pH 7.4, adjusted with KOH). Osmolarity was adjusted to 290–300 mOsm with sucrose (1.07687, Sigma‐Aldrich). Transient sodium currents were recorded in a special bath solution with a lower concentration of sodium ions.

Data acquisition was performed using pCLAMP 10.0 software with a Digidata 1322A acquisition system (Molecular Devices). Signals were low‐pass filtered at 2 kHz, sampled at 10 kHz, and analyzed offline. To analyze membrane activity properties, membrane potential was held at −60 mV in the current clamp mode. The threshold for APs was detected by applying a depolarizing current step of 5 ms duration and 20 pA increment. Additional dynamics of single spikes, such as latency of the first spike and discharge frequency, were measured with a current step of 500 ms duration and 50 pA increment. AP threshold was determined by differentiating the AP waveform and setting the inflection point at a rise rate of 10 mV/ms. AP amplitude was measured from the threshold to the peak value. AP duration was measured at half‐width of the spike. The rise and decay slopes from the AP threshold to the peak were detected.

### 2.24 Statistical analysis

The data were analyzed using R programming language version 4.3.0 and RStudio integrated development environment (IDE) for programming. The measurement data are presented as mean ± standard deviation (mean ± SD). One‐way analysis of variance (ANOVA) was performed to compare differences between groups, followed by Tukey's multiple comparison post‐test to adjust for multiple comparisons. For time‐dependent data, two‐way analysis was used with GraphPad Prism 8 software (GraphPad Software, La Jolla, CA) for graph plotting and analysis. Statistical significance was defined as a *p*‐value ≤0.05. All statistical analyses were conducted using GraphPad Prism 8 software (GraphPad Software, La Jolla, CA).

## 3 RESULTS

### 3.1 SIN and Lid regulate MMP9 to alleviate DNP treatment

Research indicates that SIN possesses analgesic and anti‐inflammatory properties, which can alleviate DNP by mitigating inflammatory reactions, improving neural blood circulation, and inhibiting neurodegeneration.^25^ Specifically, the regulation of inflammatory reactions is considered one of the essential mechanisms by which SIN alleviates pain.^26^ Lid, a local anesthetic, is commonly used for analgesic purposes. Blocking neural pathways and inhibiting the transmission of pain signals can reduce or eliminate pain perception.^27^, ^28^ In this study, we aim to develop a synergistic release formulation of SIN and Lid for the treatment of DNP, with the goal of reducing inflammation and providing long‐lasting analgesia.

**FIGURE 1:**
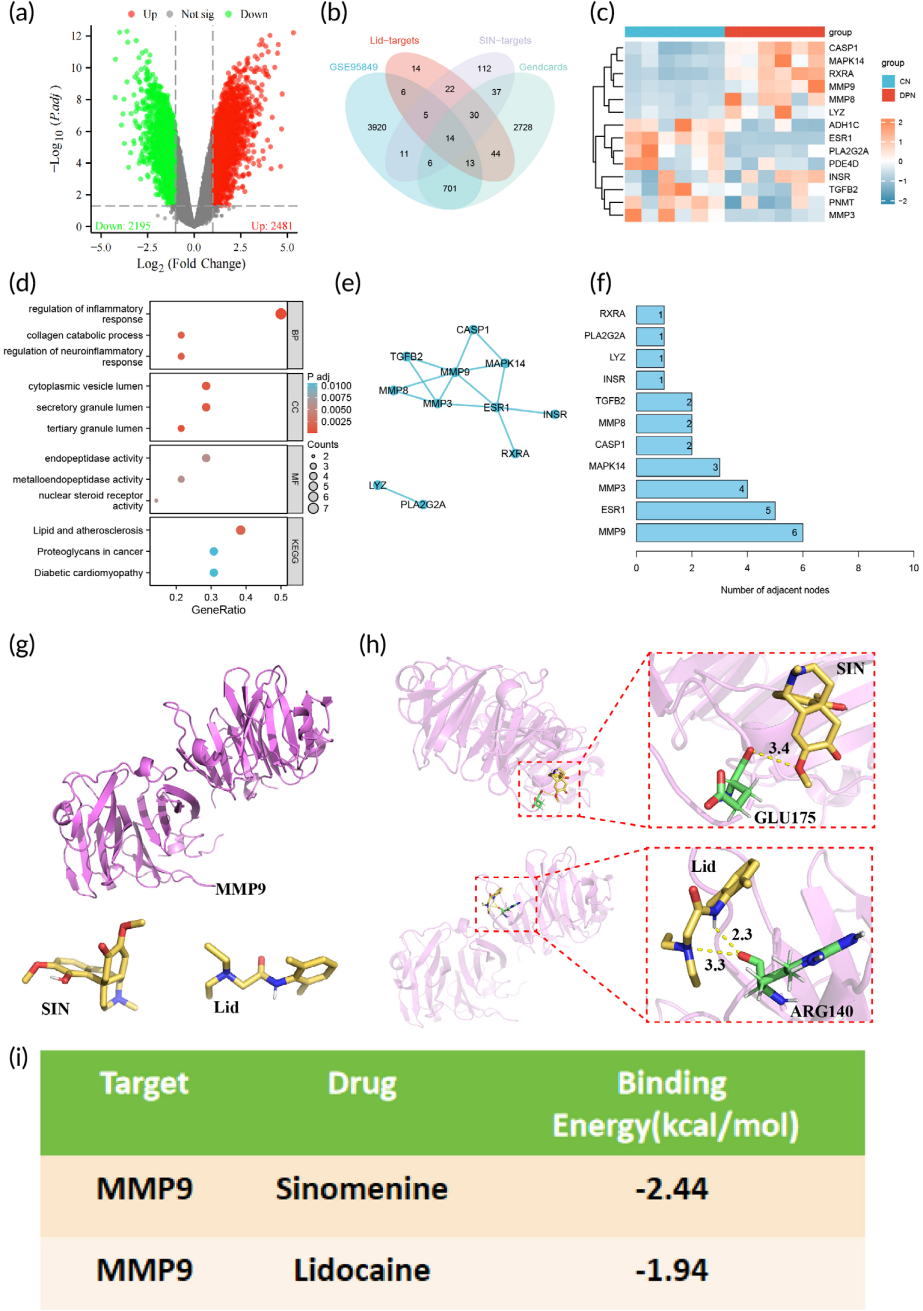
Key target gene screening of the effects of SIN and Lid on DNP. (a) Volcano plot of DEGs in GSE95849, green dots indicate downregulation, red dots indicate upregulation, and gray dots indicate no significant difference. (b) Venn diagram of target genes of SIN and Lid, as well as genes retrieved from the GEO and GeneCards databases. (c) Heatmap of differential expression of 14 key genes in GSE95849. (d) GO and KEGG enrichment analysis of the 14 key genes. (e) PPI network topology analysis of the 14 target genes involved in the progression of DNP. (f) Statistical chart of node counts in the PPI network. (g) 3D structures of MMP9 protein, SIN, and Lid. (h, i) Docking interactions and binding energy statistics between MMP9 protein with SIN and Lid molecules.

To begin, the chemical structures of SIN and Lid were obtained from the PubChem compound database. Using the PharmMapper database, 237 target genes for SIN and 148 target genes for Lid were selected. Additionally, the GEO database provided the DNP‐related GSE95849 chip dataset, which included peripheral blood samples from 6 diabetic peripheral neuropathy patients and 6 healthy control subjects. A total of 4676 DEGs were identified, comprising 2481 upregulated DEGs and 2195 downregulated DEGs (Figure 1a). DNP‐related genes were retrieved from the GeneCards database with a Relevance score ≥5, resulting in 3573 genes. Venn analysis was performed on the target genes of SIN and Lid, the DEGs from GSE95849, and the DNP‐related genes obtained from the GeneCards database, ultimately selecting 14 genes associated with DNP regulation (Figure 1b). In the dataset GSE95849, 14 key genes were identified, with 6 genes—CASP1, MAPK14, RXRA, MMP8, LYZ, and MMP9—showing significant upregulation, and nine genes—ADH1C, ESR1, PLA2G2A, PDE4D, INSR, TGFB2, PNMT, and MMP3—exhibiting significant downregulation (Figure 1c). Through GO and KEGG enrichment analyses, these genes were found to be primarily involved in critical biological processes (BP) and pathways, including the regulation of inflammatory responses, metabolic processes, vesicular and granular cell components, peptidase activity, as well as in conditions like atherosclerosis and diabetic cardiomyopathy. This suggests that SIN and Lid are closely linked to diabetes‐associated neuroinflammation and may influence neuropathic pain through the modulation of neuroinflammatory and apoptotic processes. In the BP category, the genes primarily regulate inflammatory responses and neuroinflammation, as well as collagen metabolism, indicating their key roles in tissue repair and immune response. In the Molecular Function (MF) category, they are involved in peptidase activity, metallopeptidase activity, and nuclear steroid receptor activity, highlighting their importance in protein degradation, signal transduction, and receptor regulation. Additionally, the cellular component (CC) category includes cytoplasmic vesicles and secretory granules, suggesting that SIN and Lid might impact the development of DNP at the cytoplasmic level. The KEGG enrichment results indicate that SIN and Lid may exert their biological effects by regulating lipid metabolism pathways and atherosclerosis (Figure 1d).

To explore the mechanisms behind the therapeutic effects of SIN and Lid on DNP, we imported the 14 identified targets into the STRING database to construct a PPI network (Figure 1e). Using Cytoscape software to analyze the node relationships within the PPI network, we identified MMP9 as a central node significantly associated with the network (Figure 1f). To assess the binding energies and interaction patterns between the candidate drugs/small molecules and their targets, we obtained the 3D structures of the MMP9 protein and the compounds SIN and Lid (Figure 1g). Molecular docking analyses of the candidate gene MMP9 protein with compounds SIN and Lid were conducted using software like AutoDockTools 1.2.2 and Vina 1.2.2. Molecular docking results showed that both SIN and Lid could bind to the active site of MMP9: SIN was stably embedded in the active pocket primarily through hydrogen bonding interactions, particularly with the key residue GLU175; Lid was bound mainly via hydrogen bonding with ARG140 (Figure 1h). The binding energies of SIN and Lid with MMP9 were −2.44 and −1.94 kcal/mol, respectively (Figure 1i), both less than 0 kcal/mol, indicating favorable binding. These results suggest that MMP9 may be a key target gene mediating the therapeutic effects of SIN and Lid in DNP treatment.

This analysis confirms that MMP9 is a key target gene involved in the therapeutic action of SIN and Lid against DNP.

### 3.2 Preparation and drug release behavior of temperature‐sensitive Fiber‐SIN/Gel‐LidC gel composite system

**FIGURE 2:**
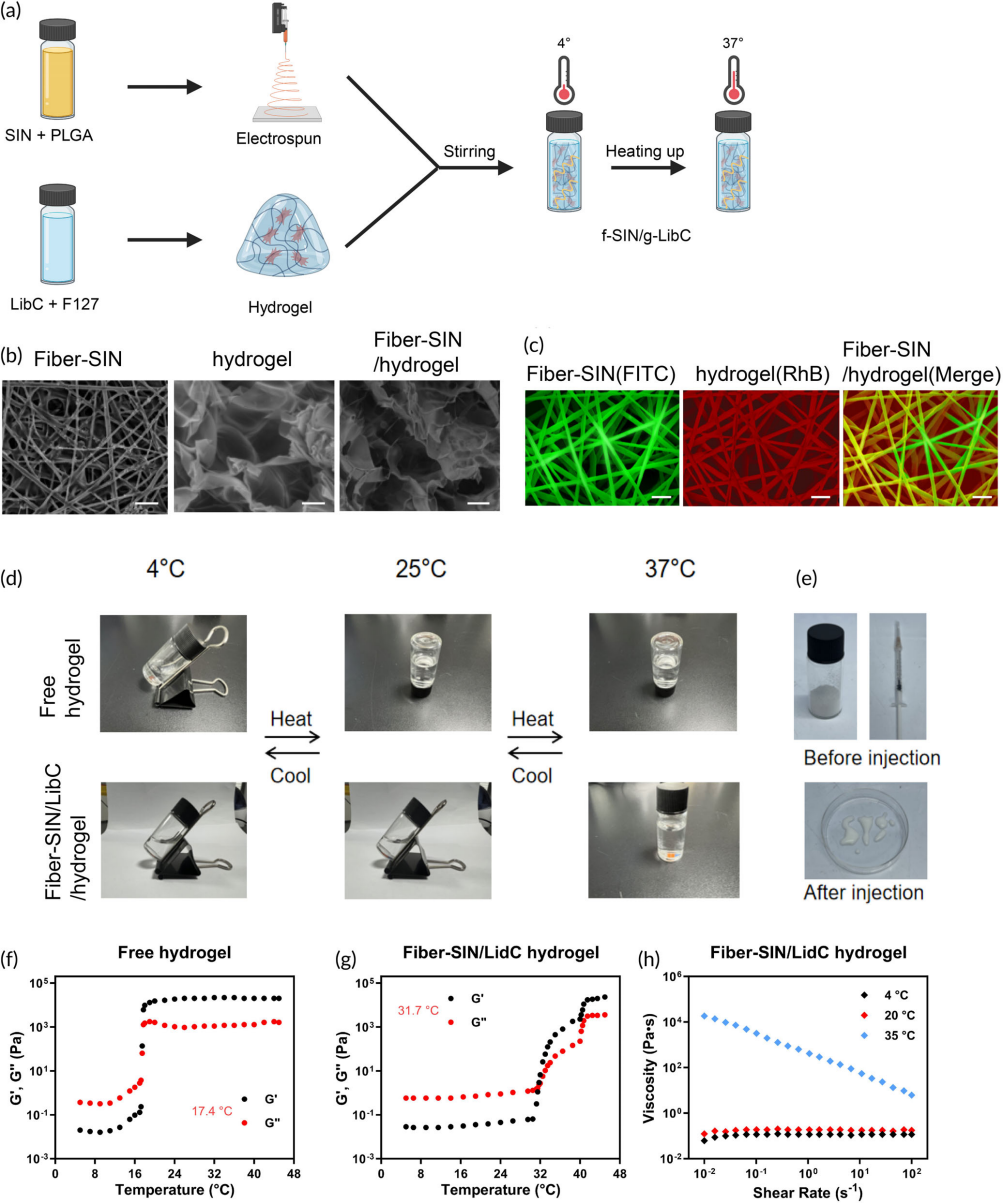
Preparation and characterization of Fiber‐SIN/Gel‐LidC. (a) Schematic diagram of the preparation of Fiber‐SIN/Gel‐LidC. (b) SEM images of Fiber‐SIN, F127 hydrogel, and Fiber‐SIN/F127 gel composite, bar = 20/50/100 μm. (c) Fluorescence microscopy images of fluorescently labeled Fiber‐SIN (FITc, green), F127 hydrogel (RhB, red), and Fiber‐SIN/F127 gel composite, bar = 100 μm. (d) Photos showing the temperature‐dependent reversible sol–gel transition of blank hydrogel and Fiber‐SIN hydrogel, the addition of Fiber‐SIN and SIN slightly increased the gelation temperature. (e) Observations on the injectability of Fiber‐SIN/Gel‐LidC. (f–g) Rheological measurements of the elastic modulus (G′) and viscous modulus (G″) of the hydrogel, (f) for blank gel, (g) for gel loaded with Fiber‐SIN/Gel‐LidC. (h) Viscosity of Fiber‐SIN/Gel‐LidC hydrogel as a function of shear rate, the system viscosity remains basically unchanged with increasing shear rate at 4–20°C. The experiment was repeated three times.

Commercial anesthetic agents are commonly provided in the form of hydrochloric acid solutions, which exhibit high water solubility but have difficulty penetrating neuronal cell membranes.^29^ Conversely, the hydrophobic free base form of drug molecules demonstrates a strong ability to penetrate and bind to hydrophobic cell membranes, which protects the drug from absorption and clearance, thus enhancing its analgesic effect.^30^, ^31^ Furthermore, the hydrophobicity of a drug affects its encapsulation efficiency and drug‐release behavior within polymers. Compared to hydrophilic drugs, highly hydrophobic drugs are more easily enveloped by the hydrophobic matrix of amphiphilic biomaterials, leading to stronger sustained release characteristics.^32^ Therefore, this experiment utilizes an anti‐solvent crystallization method to prepare LidC particles and electrospins them with amphiphilic PLGA and SIN to fabricate fiber‐SIN fibers (Figure 2a).

Lid·HCl appears as a white powder that is soluble in water. To achieve sustained release, LidC particles with different sizes were prepared using the anti‐solvent crystallization method (Figure S1a, Supporting Information). Under a stirring time of 2 hours, the obtained crystals had an average length of 20 μm (Figure S1b). Scanning electron microscopy (SEM) images show that LidC particles have a regular rod‐like shape, while Lid particles exhibit irregular crystal structures characterized by varying sizes and disordered arrangement (Figure S1b). The crystal structure of LidC remains stable after 1 month of storage at 4°C, indicating potential for long‐term preservation. X‐ray diffraction (XRD) spectra of the prepared LidC and Lid show no significant differences. LidC exhibits a strong diffraction peak at 10.68°, consistent with the diffraction pattern of Lid. No impurity peaks are detected in the XRD spectra, indicating higher purity of the prepared LidC compared to Lid (Figure S1c). Further Fourier‐transform infrared spectroscopy (FT‐IR) analysis was conducted to verify any potential changes in functional group structure during the reaction process. Lid molecules exhibit typical infrared peaks, including a peak at 3174 cm^−1^ for the N‐H bond, 2952 cm^−1^ for the C‐H bond, 1650 cm^−1^ for the carbon–oxygen double bond, 1234 cm^−1^ for the C‐N bond, and 771 cm^−1^ for aromatic sp2 C‐H groups. The spectra demonstrate that the preparation process of microcrystals does not significantly alter the chemical structure of the Lid under study, and LidC still exhibits typical absorption peaks and bands consistent with Lid (Figure S1d). Microcrystals with a length of 6 μm rapidly and completely release within 12 h, while those with a length of 20 μm exhibit zero‐order release kinetics over approximately 4 days, and microcrystals of 30 μm in length continuously release Lid throughout the entire test period (7 days), with an accumulated release of 84.3 ± 0.7% (Figure S1e–g). Based on the aforementioned research results, LidC, with a length of 20 μm, is selected as the optimal candidate material for the sustained release system.

We utilized the electrospinning method to prepare SIN sustained‐release fibers. In order to obtain the optimal electrospinning fiber formulation, a series of electrospinning parameters were fine‐tuned for PLGA (Table S1). By adjusting and optimizing the mass ratio of PLGA to SIN, solution injection rate, voltage, and solvent, we achieved stable web‐like fibers. SEM observation revealed that the PLGA electrospun fibers exhibited smooth thread‐like structures without noticeable beading (see Figure S2a). The average diameter of the blank PLGA electrospun fibers was measured to be 5.4 ± 1.0 μm (Figure S2b), whereas Fiber‐SIN had an average diameter of 2.42 ± 0.84 μm (Figure S2c). This may be attributed to the change in the coiling degree of the PLGA molecular chains caused by the addition of SIN.

To achieve a highly injectable, minimally invasive drug delivery gel system, we tested F127 solutions with different mass compositions and found that a 40% F127 hydrogel (wt%) was the ideal choice (Table S2). We fully immersed 28 mg of Fiber‐SIN and 10 g of deionized water in 1 mL of the F127 solution and prepared the Fiber‐SIN/Gel composite at 37°C. The gel composite was successfully injected through a 1.2‐mm‐diameter needle (Figure S3), indicating its suitability for local injection therapy.

SEM observation was performed on the dual‐loaded Fiber‐SIN/Gel‐LidC composite material for injectable drugs. In the gel composite system, the diameter of the Fiber‐SIN fibers slightly contracted, transforming into short fibers with a cotton‐like appearance (Figures 2b and S1d), which facilitated gel injection. Using fluorescence microscopy, we clearly observed the two components: Fiber‐SIN and F127 hydrogel.

To investigate the dispersion of Fiber‐SIN fibers and F127 hydrogel in the injectable gel composite material (Figure 2c), we observed that the fibers were well‐dispersed in the gel, and the drug encapsulated within the PLGA fibers did not diffuse into the gel phase. At 4°C, the temperature‐sensitive F127 was in a sol state, allowing the Fiber‐SIN fibers and SIN drug soaked in it to disperse well, which then transitioned to a gel phase at 37°C. With the addition of Fiber‐SIN fibers and SIN drug, the gelation temperature of F127 increased and remained in sol state at 25°C, facilitating room temperature injection (Figure 2d).

The Fiber‐SIN and SIN drug in the Fiber‐SIN/Gel‐LidC composite material readily transformed from a sol state to a gel state (Figure 2e), as further confirmed by rheological analysis. The gelation temperatures of the blank gel and drug‐loaded gel complex were 17.4 and 31.7°C, respectively (Figure 2f,g). Under conditions of 4–20°C, the viscosity of the drug‐loaded gel complex was low (approximately 0.18 Pa·s) and remained unchanged with increasing shear rate. This favorable flowability is advantageous for the preparation and application of the hydrogel. When the temperature increased to 35°C, the viscosity of the drug‐loaded gel complex rapidly decreased with increasing shear rate, dropping from 17,600 Pa to 6.31 Pa, demonstrating shear‐thinning behavior (Figure 2h). This study further explored the therapeutic effects of the Fiber‐SIN/Gel‐LidC composite on DNP and attempted to distinguish the roles of the material and the drug components. The hydrogel material alone (DNP + Gel group) showed no analgesic effect, while the combination of SIN and Lid (DNP + SIN + Lid group) produced moderate analgesic and anti‐inflammatory effects without the support of the material. Notably, the complete composite (DNP + Fiber‐SIN/Gel‐LidC group) demonstrated more sustained analgesia, suggesting that the material may enhance therapeutic effects through prolonged drug release, improved tissue permeability, or increased bioavailability (Figure 2i). This finding highlights the critical role of drug delivery carriers in DNP treatment and provides valuable guidance for future material optimization.

**FIGURE 3:**
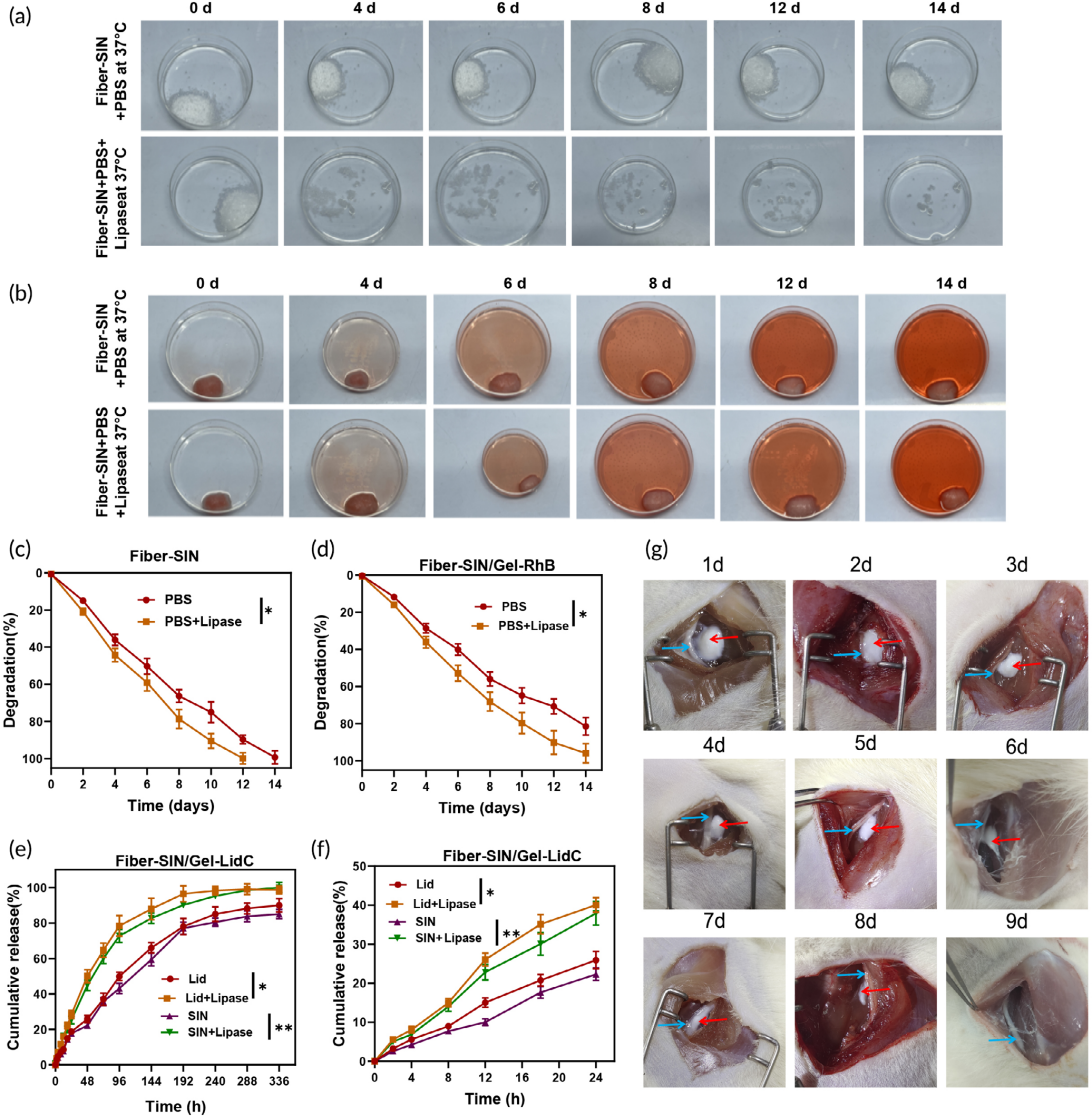
Degradation and release study of Fiber‐SIN/Gel‐LidC. (a) Morphological photos of Fiber‐SIN in PBS solution with or without lipase (20 U/mL) for 0–14 days. (b) The degradation of Fiber‐SIN/Gel‐RhB in PBS with or without 20 U/mL lipase was recorded to assess material stability and enzymatic responsiveness. (c) Degradation rate curve of Fiber‐SIN. (d) Morphological photos of Fiber‐SIN/Gel‐RhB composite material in PBS solution with or without lipase (20 U/mL). (e, f) Release behavior of Fiber‐SIN/Gel‐LidC gel in PBS solution with or without lipase (20 U/mL), (e) cumulative release curve for 14 days, (f) zoomed‐in view of cumulative release curve for 24 h. (g) Local anatomical structures of the sciatic nerve and surrounding muscles, as well as potential composite residues, captured at different time points after injection. The cyan arrow indicates the sciatic nerve, and the red arrow indicates gel residue, bar = 1 cm. The data at different time points were analyzed using a two‐way ANOVA. The in vitro experiment was repeated three times, and the animal experiment had six animals per group (*n* = 6), **p < 0.01, *p < 0.05.

To determine the drug release timeline further, we studied the degradation and drug release behavior of the Fiber‐SIN/Gel‐LidC composite gel. Through in vitro experiments, we observed the degradation and release behavior of the drug‐loaded Fiber‐SIN/Gel‐LidC system. The loss in weight due to Fiber‐SIN fiber degradation was 24% and 52% within 6 days in PBS with and without Lipase, respectively (Figure 3a,b). To facilitate the observation of gel degradation, we added RhB. In the presence of Lipase in PBS, the gel completely degraded within 14 days, leaving behind only Fiber‐SIN fibers (Figure 3c,d). In the PBS solution, the gel system showed slow release of Lid, reaching release percentages of 2.1 ± 0.4%, 7.8 ± 0.3%, 23.4% ± 0.8%, and 86.1 ± 0.5% at 6 h, 10 h, 24 h, and 12 days, respectively. The release of SIN from the gel system continued for approximately 14 days, but the release rate increased when Lipase was added, indicating that the enzymatic environment facilitated gel degradation (Figure 3e,f). In the PBS + Lipase group, the accelerated release of Lid further demonstrated that enzymatic hydrolysis promotes drug release (Figure 3f). In the complex and enzymatic‐rich in vivo environment, we observed the amount of residual material after the injection of the gel system. The results showed only a small amount of residue on the 8th day (Figure 3g). Considering the effective drug concentration, we set the injection interval to 7 days. In summary, we successfully developed an injectable, temperature‐sensitive, high drug‐loading Fiber‐SIN/Gel‐LidC gel composite system with sustained drug release behavior, providing potential for long‐term local pain treatment.

### 3.3 Alleviating DNP through ion channel modulation using Fiber‐SIN/Gel‐LidC

Bioinformatics analyses earlier demonstrated that MMP9 is a crucial target gene playing a key role in the treatment of DNP with SIN and Lid. In this study, we successfully synthesized a composite material, Fiber‐SIN/Gel‐LidC, which exerts synergistic sustained‐release effects. We intend to further investigate the therapeutic effects of this composite material on DNP treatment. Initially, we established a rat model of DNP. Compared to normal rats (with blood glucose levels at 90.53 ± 1.56 mmol/L), the STZ‐induced diabetic rats showed significantly elevated blood glucose levels (421.67 ± 3.61 mmol/L). Additionally, the weight of diabetic rats (221.33 ± 3.76 g) decreased when compared to normal rats (281 ± 4.08 g) (Table S3). We observed a significant decrease in the heat threshold of diabetic rats over time, as evidenced by the measurement of thermal threshold and mechanical withdrawal threshold (Figure S4a). Similarly, the mechanical withdrawal threshold test indicated downregulation of the threshold due to diabetes (Figure S4b). These results confirm the successful establishment of the DNP model.

**FIGURE 4:**
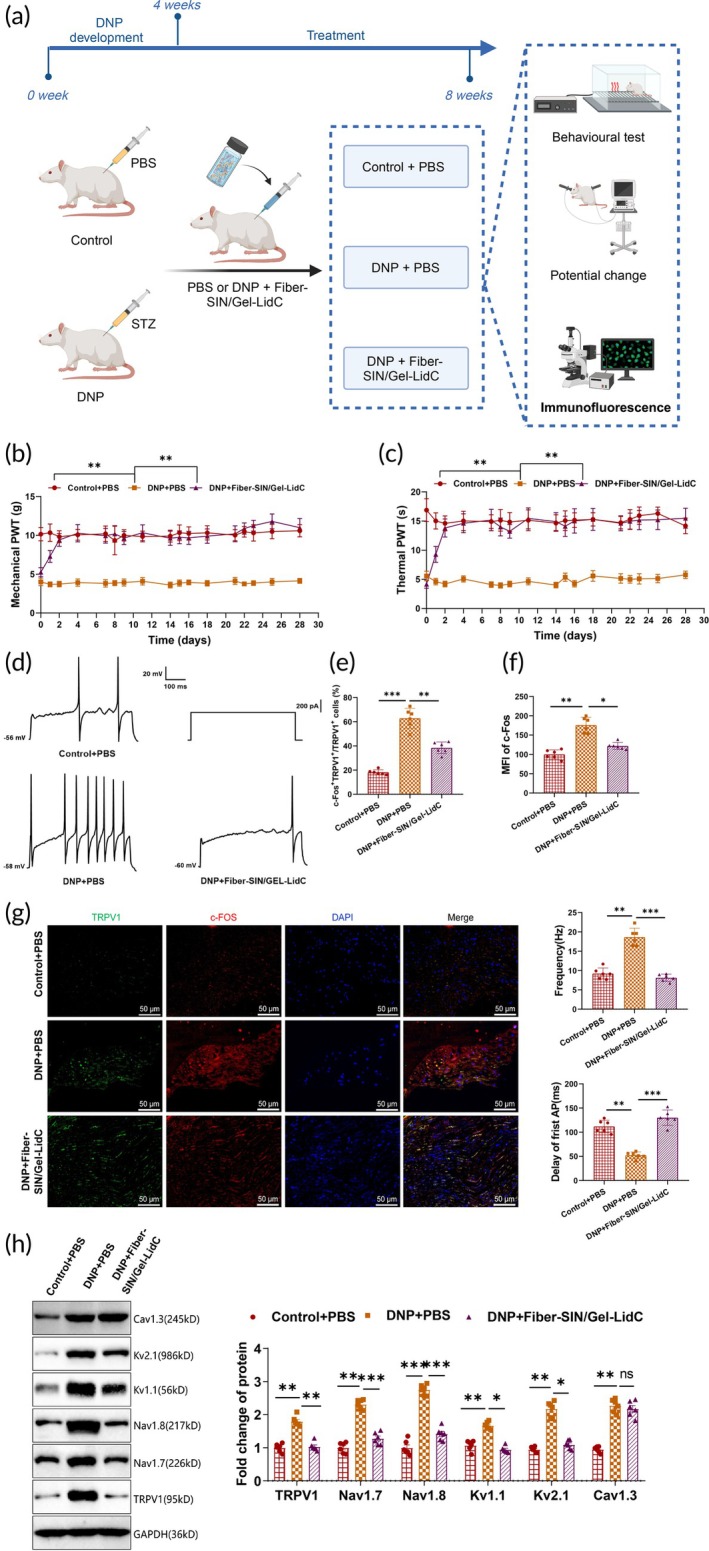
Experimental validation of Fiber‐SIN/Gel‐LidC in inhibiting neural excitation and reducing neural pain. (a) Schematic representation of experimental treatment. (b, c) Changes in pain threshold in rats treated with Fiber‐SIN/Gel‐LidC over 2 weeks; bar graphs divided into 100 μm. (d) Representative trace plots of peak discharge in small DRG neurons of different treatment groups in response to depolarizing current steps. (e, f) Statistical graphs showing the firing frequency (e) and AP peak latency (f) of DRG neurons in different treatment groups. (g) Immunofluorescence examples of DRG, c‐Fos (red) and TRPV1 (green), DAPI (blue) stained nucleus, bar graphs divided into 100 μm. (h) Expression of neural excitatory voltage‐gated ion channel proteins in different treatment groups. Multiple group comparisons were conducted using one‐way ANOVA, while data from different time points were analyzed using two‐way ANOVA. Each group consisted of six rats (*n* = 6), ***p < 0.001, **p < 0.01, *p < 0.05.

To evaluate the impact of Fiber‐SIN/Gel‐LidC on DNP, we initiated the intrathecal injection of Fiber‐SIN/Gel‐LidC gel on rats from the 5th week after STZ injection. The gel was administered once every 7 days for a total of 4 cycles. Through behavioral testing, neural electrophysiology, and immunofluorescence, we evaluated the analgesic effects of Fiber‐SIN/Gel‐LidC gel (Figure 4a). Following treatment with Fiber‐SIN/Gel‐LidC gel in diabetic rats, the mechanical pain threshold and thermal pain threshold significantly increased and remained relatively stable, attributed to the slow and steady release of SIN and Lid (Figure 4b,c).

Further investigation was conducted to explore the mechanisms by which Fiber‐SIN/Gel‐LidC alleviates DNP. The effects of Fiber‐SIN/Gel‐LidC on the membrane characteristics and excitability of DRG neurons in DNP rats were analyzed. Compared to the Control + PBS group, significant differences in the active membrane characteristics of DRG neurons were observed in the DNP + PBS group (Table S4). Increased excitability was recorded in small DRG neurons in the DNP + PBS group, characterized by increased action potential amplitude, shorter action potential duration, increased action potential slope, and decreased action potential threshold. However, there were no differences in the passive membrane characteristics of DRG neurons between the Control + PBS group and the DNP + PBS group (Table S4). After four cycles of Fiber‐SIN/Gel‐LidC treatment, the excitability of small DRG neurons in the DNP + PBS group was significantly reduced (Table S4). Importantly, the average discharge frequency in response to depolarizing current steps was significantly higher in small DRG neurons of the DNP + PBS group compared to the Control + PBS group (Figure 4d–f). This rigid firing frequency could be significantly reduced with Fiber‐SIN/Gel‐LidC treatment. Further analysis of the first peak latency revealed that the first peak latency evoked by the same intensity of stimulation was significantly shorter in the DNP + PBS group compared to the Control + PBS group. In contrast, the first peak latency in the DNP + Fiber‐SIN/Gel‐LidC group was prolonged compared to the DNP + PBS group, indicating reduced neuronal excitability (Figure 4d–f). Painful neuropathy is mediated by primary sensory neurons located in the trigeminal ganglia and DRG. These neurons transmit pain signals to the brainstem or spinal dorsal horn, which then propagate to central circuits, thalamus, and higher cortical centers, ultimately leading to pain perception.^33^, ^34^ Transient receptor potential cation channel subfamily V member 1 (TRPV1) is highly expressed in the trigeminal ganglia and DRG and serves as a specific molecular marker for nociceptive neurons.^35^, ^36^ Activation of DRG neurons was studied using immunofluorescent staining of c‐Fos and TRPV1 to investigate pain pathways.^37^, ^38^, ^39^ Compared to the Control + PBS group, activation of TRPV1‐positive neurons was significantly increased in the DNP + PBS group. Fiber‐SIN/Gel‐LidC treatment was able to suppress the activation of primary sensory neurons in the DRG, exerting analgesic effects (Figure 4g,h).

Previous studies have reported that SIN and Lid have sedative and analgesic effects by modulating neuronal excitability‐gated channels.^40^, ^41^, ^42^ Testing was done on sodium, potassium, and calcium ion‐related proteins associated with neuronal excitability‐gated channels. Compared to the Control + PBS group, the expression of neuronal excitability‐gated channel‐related proteins was significantly increased in the DNP + PBS group. After Fiber‐SIN/Gel‐LidC treatment, significant reductions were observed in Nav1.7 and Nav1.8 proteins associated with sodium ion‐gated channels, as well as Kv1.1 and Kv2.1 proteins associated with potassium ion‐gated channels, with more significant changes in sodium ion‐gated channel‐related proteins. There were no significant changes in Ca1.3 protein associated with calcium ion‐gated channels. These results indicate that Fiber‐SIN/Gel‐LidC treatment can significantly inhibit sodium ion and potassium channel activity, thus alleviating pain (Figure 4h).

In conclusion, the above experimental results demonstrate that Fiber‐SIN/Gel‐LidC can alleviate DNP by downregulating sodium channel and potassium channel activity.

### 3.4 Fiber‐SIN/Gel‐LidC exerts neuroprotective effects by modulating the neuroinflammatory process

Previous studies have demonstrated the therapeutic effects of Fiber‐SIN/Gel‐LidC on neuropathic pain from a pain‐suppression perspective; however, the specific mechanisms of its action on nerves have not been investigated. Activation of the neuroimmune gate has a close relationship with inflammation.^43^ Existing literature has reported the involvement of MMP9 in neuronal damage, inflammation mediation, and regulation of blood–brain barrier permeability. This protease can degrade extracellular matrix molecules, regulate neurotransmitter release, and modulate the expression of cell adhesion molecules.^44^, ^45^ Studies have indicated that increased MMP9 activity is closely associated with the inflammatory process, potentially leading to cell death and infiltration of inflammatory cells.^46^, ^47^ The preceding experimental analysis revealed that MMP9 is the main target of Fiber‐SIN/Gel‐LidC in the treatment of DNP. Thus, the objective of this study is to investigate the impact of Fiber‐SIN/Gel‐LidC treatment on neuroinflammation.

**FIGURE 5:**
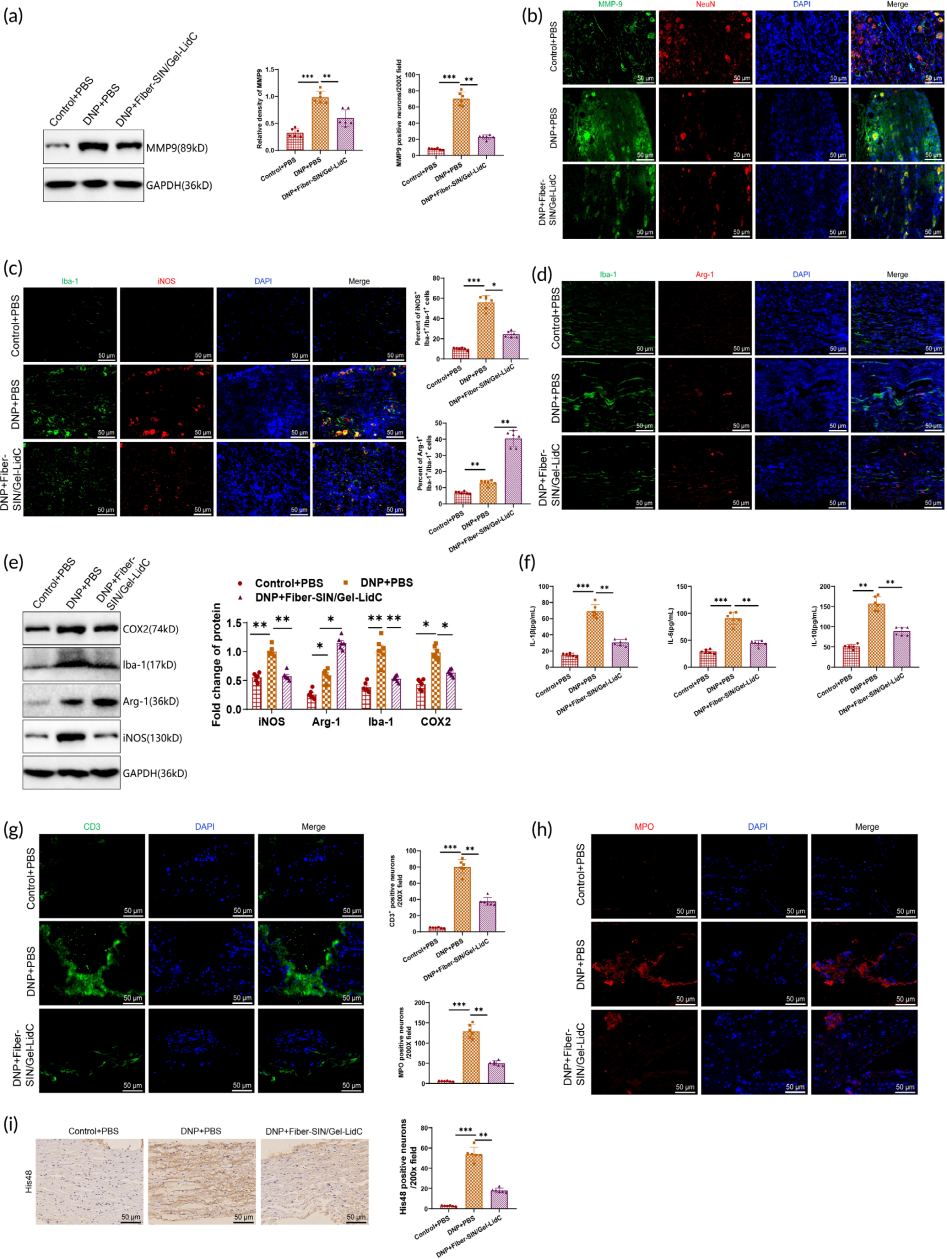
Experimental validation of Fiber‐SIN/Gel‐LidC in inhibiting DNP‐induced neural inflammation. (a) Representative protein bands and quantitative graphs of relative MMP9 expression by Western blot. (b) Representative micrographs and quantitative graphs of matrix metalloproteinase 9 (MMP9, green) positive neurons (red), bar graph = 100 μm. (c) Representative immunofluorescence images of iNOS positive neurons (red, M1 phenotype) in macrophages, bar graph = 100 μm. (d) Representative immunofluorescence images of Arg1 positive neurons (red, M2 phenotype) in macrophages in small neurons, bar graph = 100 μm. (e) Representative protein bands and quantitative graphs of COX‐2, Iba‐1, Arg‐1, and iNOS (*n* = 6) expression. (f) ELISA detection of IL‐1β, IL‐6, and IL‐10 levels in different groups. (g) Representative immunofluorescence images and quantitative graphs of CD3 positive neurons (green), bar graph = 100 μm. (h) Representative immunofluorescence images and quantitative graphs of MPO positive cells (red), bar graph = 100 μm. (i) Representative immunohistochemical images and quantitative graphs of His48 positive cells, where the arrow indicates the position of positive cells, bar graph = 100 μm. Multiple group comparisons were conducted using one‐way ANOVA. Each group consisted of six rats (*n* = 6), ***p < 0.001, **p < 0.01, *p < 0.05.

MMP9 expression differences were compared among the Control + PBS group, DNP + PBS group, and DNP + Fiber‐SIN/Gel‐LidC group using Western blot analysis. Compared to the Control + PBS group, MMP9 expression was significantly elevated in the DNP + PBS group, which is consistent with the previous bioinformatics results. However, MMP9 expression in the DNP + Fiber‐SIN/Gel‐LidC group was reduced compared to the DNP + PBS group (Figure 5a). The proportion of MMP9‐positive cells in the DRG of different groups was quantified through fluorescence staining. Compared to the Control + PBS group, the number of MMP9‐positive cells was significantly increased in the DNP + PBS group but decreased in the DNP + Fiber‐SIN/Gel‐LidC group (Figure 5b).

To assess neuroinflammation, immunostaining of iNOS and Arg‐1 was performed on the DRG (PMID: 37115404, PMID: 30739804, PMID: 23738033). As expected, the number of iNOS‐positive and Arg‐1‐positive cells in the DNP + PBS group was significantly higher than in the Control + PBS group, indicating a prominent inflammatory reaction in the DNP + PBS group. After treatment with Fiber‐SIN/Gel‐LidC, the DNP + Fiber‐SIN/Gel‐LidC group exhibited a reduction in iNOS‐positive cells and an increase in Arg‐1‐positive cells compared to the DNP + PBS group (Figure 5c,d). Furthermore, Western blot analysis showed that the protein levels of Iba‐1 and iNOS were significantly lower, while the level of Arg‐1 was significantly higher in the DNP + Fiber‐SIN/Gel‐LidC group compared to the DNP + PBS group (Figure 5e).

Cyclooxygenase‐2 (COX‐2) plays a crucial role in neuroinflammation.^48^ Protein immunoblot analysis demonstrated that COX‐2 expression was significantly increased in the DNP + PBS group, while Fiber‐SIN/Gel‐LidC effectively reduced COX‐2 expression (Figure 5e). Furthermore, enzyme‐linked immunosorbent assay (ELISA) results showed that the expression levels of pro‐inflammatory factors (IL‐1β and IL‐6) and an anti‐inflammatory factor (IL‐10) were significantly higher in the DNP + PBS group compared to the Control + PBS group. Fiber‐SIN/Gel‐LidC downregulated the expression of IL‐1β and IL‐6 induced by DNP and upregulated the expression of the anti‐inflammatory factor IL‐10 (Figure 5f), indicating that Fiber‐SIN/Gel‐LidC can downregulate the expression of pro‐inflammatory factors while activating and upregulating the expression of anti‐inflammatory factors.

Furthermore, the infiltration of peripheral immune cells was evaluated by staining for CD3 (T lymphocytes), MPO (neutrophils), and His48 (granulocytes). Immunofluorescence staining and immunohistochemical results showed that the counts of T lymphocytes, neutrophils, and granulocytes in the DNP + PBS group were higher than in the Control + PBS group. Moreover, Fiber‐SIN/Gel‐LidC significantly reduced the number of peripheral immune cells (Figure 5g–i).

In summary, the results of the experiments above demonstrate that Fiber‐SIN/Gel‐LidC exerts neuroprotective effects by modulating the immune system to alleviate the inflammatory reaction.

### 3.5 Regulation of high glucose‐induced cell apoptosis and neuroprotection by Fiber‐SIN/Gel‐LidC

**FIGURE 6:**
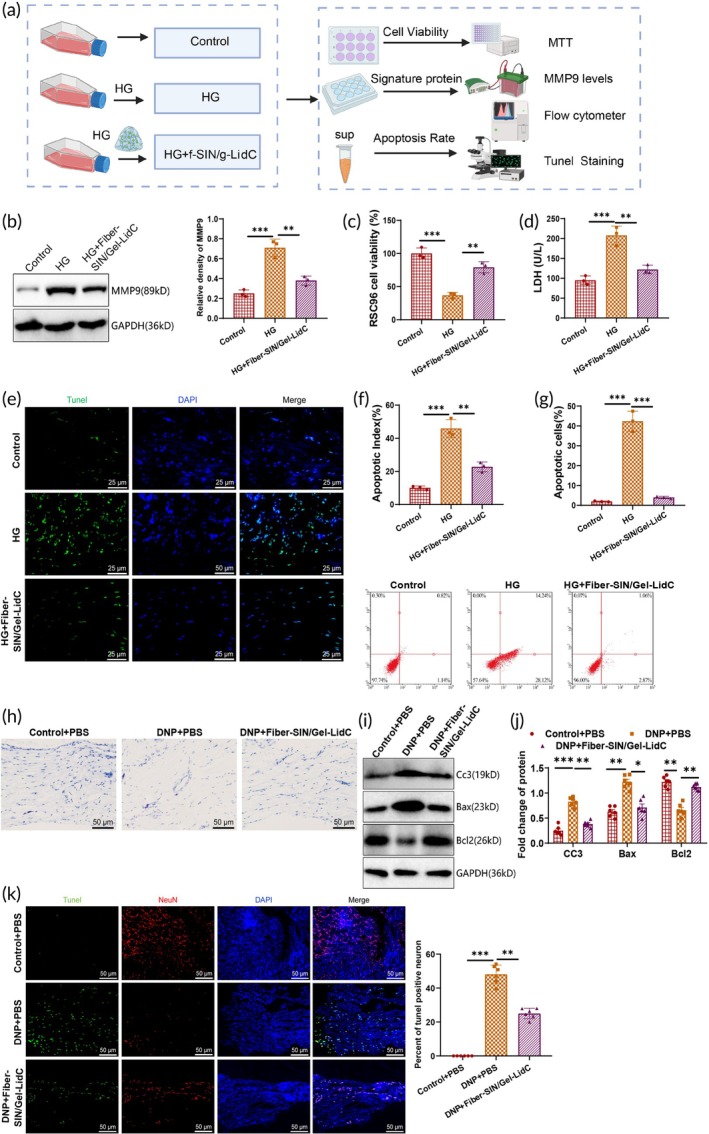
Experimental validation of Fiber‐SIN/Gel‐LidC in downregulating MMP9 expression and reducing neural apoptosis induced by diabetic neuropathy. (a) Schematic representation of cell experiments. (b) Representative protein bands and quantitative graphs of relative MMP9 expression by Western blot. (c) Cell viability of different treatment groups measured by MTT assay. (d) LDH release from cells in different treatment groups detected using LDH assay kit. (e) TUNEL staining (magnification 400×) to detect cell apoptosis, green represents TUNEL positive apoptotic cells, blue represents DAPI stained cell nucleus. (f) Quantitative statistics of cell apoptotic rate. (g) Flow cytometry analysis of early cell apoptotic rate in different treatment groups. (h) Representative micrographs of Nissl stained neurons, red arrows indicating disintegrated Nissl bodies indicating cell apoptosis, bar graph = 50 μm. (i, j) Representative protein bands (i) and quantitative graphs of Bcl‐2, Bax, and cleaved‐caspase 3 relative protein expression (j). (k) Representative micrographs and quantitative graphs of TUNEL positive neurons, bar graph = 100 μm. Multiple group comparisons were conducted using one‐way ANOVA. Cell experiments were repeated three times, and each animal experiment group consisted of six rats (*n* = 6), ***p < 0.001, **p < 0.01, *p < 0.05.

Elevated glucose levels can trigger a series of cellular stress responses, including mitochondrial dysfunction, oxidative stress, and activation of cell apoptosis signaling pathways. Activation of these signaling pathways leads to neuronal apoptosis and loss of function. MMP9 is closely associated with pathological neuronal cell apoptosis.^49^, ^50^ To observe cell behavior, RSC96 cells were treated with high glucose (HG) with or without the addition of Fiber‐SIN/Gel‐LidC (Figure 6a). Western blot analysis revealed that MMP9 expression was significantly increased in the HG group compared to the Control group, while MMP9 expression was relatively lower in the HG + Fiber‐SIN/Gel‐LidC group (Figure 6b). Cell viability results indicated that the cell viability of RSC96 cells treated with HG significantly decreased; however, treatment with Fiber‐SIN/Gel‐LidC significantly improved the cell viability of RSC96 cells treated with HG (Figure 6c). To determine if the decrease in cell viability was due to HG‐induced cell death, the LDH levels of RSC96 cells were measured, showing that HG treatment significantly increased LDH release from RSC96 cells, while Fiber‐SIN/Gel‐LidC inhibited LDH release (Figure 6d). These results suggest that Fiber‐SIN/Gel‐LidC attenuates the inhibitory effect of HG on the cell viability of RSC96 cells by downregulating MMP9 expression, and the improvement in cell viability may be related to the protective effect of Fiber‐SIN/Gel‐LidC on HG‐induced cell death.

TUNEL staining and flow cytometry were used to assess the effect of Fiber‐SIN/Gel‐LidC on apoptotic cell death in cultured cells to determine if Fiber‐SIN/Gel‐LidC affects cell viability by modulating cell apoptosis. RSC96 cells treated with HG exhibited significantly increased apoptosis compared to the Control group, while treatment with Fiber‐SIN/Gel‐LidC alleviated HG‐induced cell apoptosis in RSC96 cells (Figure 6e,f). Flow cytometry results showed that Fiber‐SIN/Gel‐LidC also suppressed early apoptosis induced by HG (Figure 6g). These results demonstrate that Fiber‐SIN/Gel‐LidC has an inhibitory effect on cell apoptosis of RSC96 cells under HG conditions.

The neuronal apoptosis in DNP rats treated with Fiber‐SIN/Gel‐LidC was further examined. Nissl staining demonstrated that the apoptotic fraction of neurons in the DNP + PBS group was significantly higher than that in the Control + PBS group. However, the cell apoptosis fraction in the DNP + f‐SIN/g‐LidC group was significantly lower than that in the DNP + PBS group (Figure 6h). Western blot analysis revealed a significant upregulation of apoptosis factors in the peripheral nerves of the DNP + PBS group. In contrast, the levels of cleaved caspase‐3 and Bax were lower, and the level of the anti‐apoptotic factor Bcl‐2 was higher in the DNP + f‐SIN/g‐LidC group (Figure 6i,j). Furthermore, the number of TUNEL‐positive neurons in the DNP + f‐SIN/g‐LidC group was significantly lower than that in the DNP + PBS group (Figure 6k). These findings indicate that f‐SIN/g‐LidC treatment can reduce neuronal apoptosis and exert neuroprotective effects following DNP.

Thus, it can be concluded that f‐SIN/g‐LidC therapy can reduce neuronal apoptosis in DNP by downregulating MMP9 and providing neuroprotection.

### 3.6 Protection of the sciatic nerve against diabetic neuropathy‐associated morphological damage by Fiber‐SIN/Gel‐LidC

After four treatment cycles, we observed the biocompatibility and local therapeutic effects of Fiber‐SIN/Gel‐LidC treatment through biochemical markers and tissue staining. The biochemical markers revealed no significant differences in liver function (Figure S5a,b) or kidney function (Figure S5c) between DNP + f‐SIN/g‐LidC rats and the DNP + PBS group. The blood routine results showed no significant differences in white blood cell and platelet counts (Figure S5d,e) between DNP + f‐SIN/g‐LidC rats and the DNP + PBS and Control + PBS groups. Additionally, no notable histopathological changes were observed in the organ examinations (Figure S5f), confirming the high biocompatibility of Fiber‐SIN/Gel‐LidC gel injections.

**FIGURE 7:**
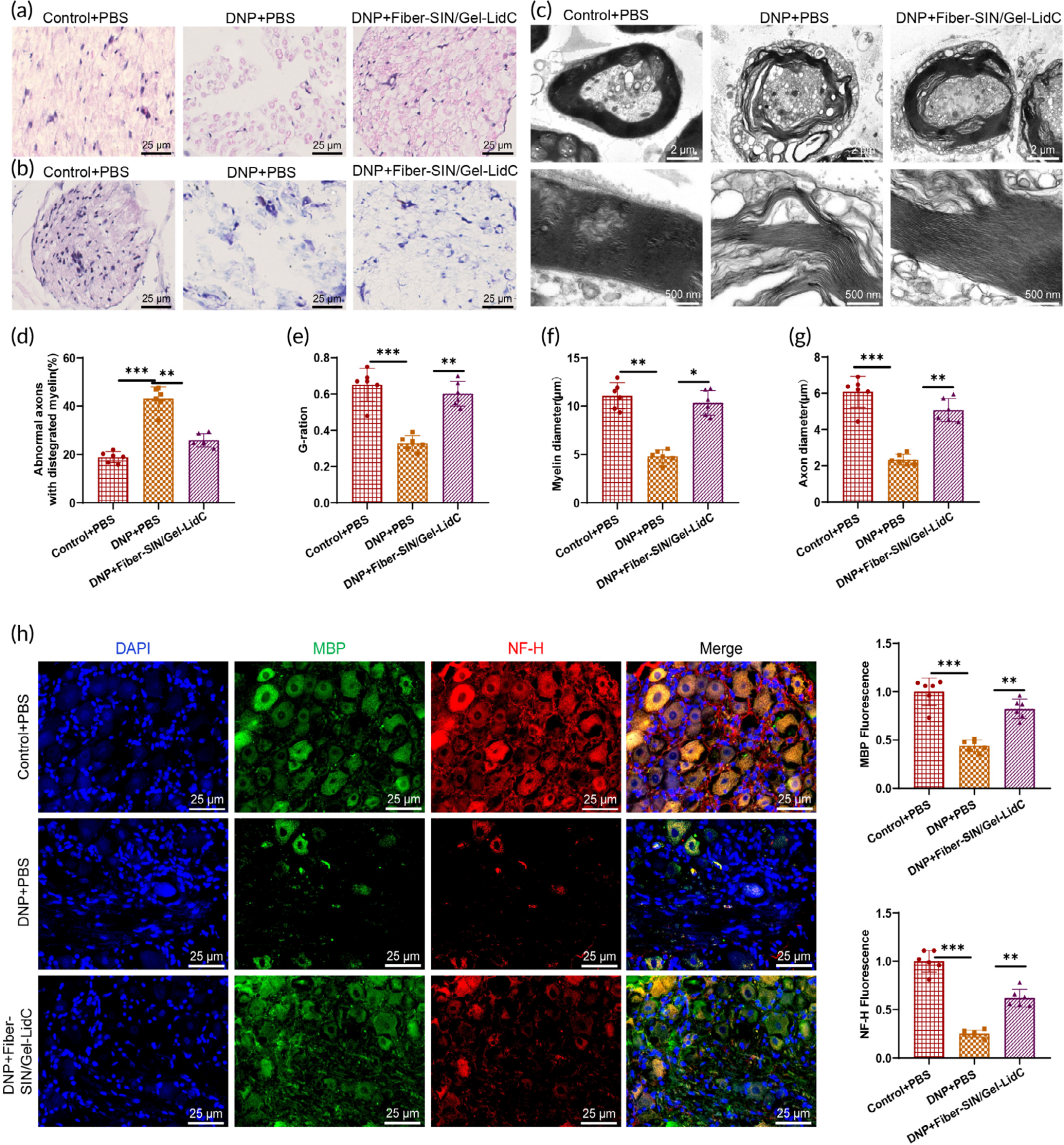
Improvement of peripheral nerve function and alleviation of peripheral nerve morphological changes in DNP rats by Fiber‐SIN/Gel‐LidC. (a, b) Morphology of the sciatic nerve in rats observed under H&E staining, with the upper scale marker = 10 μm and the lower scale marker = 25 μm. The red arrows indicate demyelination accompanied by severe vacuolation and axonal atrophy. (c) Ultrastructural observations of the sciatic nerve in rats using transmission electron microscopy, with the upper scale marker = 2 μm and the lower scale marker = 500 nm. (d–g) Statistical graphs of calculated measurements of nerve morphology parameters, including demyelination (d), abnormal axonal ratio (axonal diameter/myelin diameter) (e), myelin diameter (f), and axonal diameter (g). (h) Representative images of immunofluorescent staining and statistical graph of MBP and neurofilament‐H (NF‐H) positive cells, with the scale marker = 25 μm. Multiple group comparisons were conducted using one‐way ANOVA. Each experimental group consisted of six animals (*n* = 6), ***p < 0.001, **p < 0.01, *p < 0.05.

H&E and toluidine blue staining revealed morphological changes in the sciatic nerves of the Control + PBS group rats. The sciatic nerve exhibited a clear structure, orderly arrangement of nerve fibers, and normal morphology of myelin sheaths and axons. In the DNP + PBS group rats, the sciatic nerve showed significant demyelination and axonal atrophy. However, the DNP + Fiber‐SIN/Gel‐LidC group rats exhibited improved remyelination and reduced axonal atrophy (Figure 7a,b). Transmission electron microscopy observations revealed clear ultrastructural layers of myelin sheath fibers in the sciatic nerves of the Control + PBS group rats. In contrast, the sciatic nerves of the DNP + PBS group rats displayed evident vacuolar defects, laminar separation of myelin sheaths, and axonal atrophy. Fiber‐SIN/Gel‐LidC treatment ameliorated these defects (Figure 7c). Morphometric analysis demonstrated a significant increase in the percentage of abnormal axons in the DNP + PBS group compared to the Control + PBS group, while the DNP + Fiber‐SIN/Gel‐LidC group exhibited a notable decrease in the percentage of abnormal axons (Figure 7d). Furthermore, compared to the Control + PBS group rats, the DNP + PBS group rats showed significant reductions in G‐ratio, myelin sheath diameter, and axon diameter (Figure 7e–g). Conversely, the DNP + Fiber‐SIN/Gel‐LidC group rats exhibited a partial recovery of G‐ratio, myelin sheath diameter, and axon diameter compared to the DNP + PBS group rats. Demyelination, demyelinating glial cells, and axonal atrophy are characteristic features of peripheral nervous system disorders. Myelin basic protein (MBP) is a vital component of myelin sheath structure^51^ and plays a crucial role in the formation of nerve fiber myelin sheaths.^52^ Neurofilament heavy chain (NF‐H) is a marker associated with axonal atrophy.^53^ Therefore, we further assessed the expression of MBP and NF‐H in the DRG through immunofluorescence. MBP is primarily distributed in the myelin sheaths, while NF‐H exhibits high expression in neuronal cell bodies and axons. Compared to the Control + PBS group rats, the DNP + PBS group rats showed reduced expression of MBP and NF‐H in the DRG, indicating demyelination and regeneration impairment. However, Fiber‐SIN/Gel‐LidC treatment increased the expression of MBP and NF‐H (Figure 7h), suggesting that Fiber‐SIN/Gel‐LidC treatment facilitates the repair and regeneration of myelin sheaths and axons induced by DNP.

In conclusion, Fiber‐SIN/Gel‐LidC significantly improved these morphological alterations in the sciatic nerve, thus indicating its potential to protect against morphological damage associated with diabetic neuropathy.

## 4 DISCUSSION

In this research, the newly developed Fiber‐SIN/Gel‐LidC composite material demonstrated significant therapeutic efficacy, contrasting sharply with traditional drug delivery systems used in prior studies. Compared to single‐drug treatments, our composite material achieves synergistic release of SIN and Lid, showing advantages in both efficacy and safety. Historically, while multiple drug use strategies have been explored, few studies have utilized the combination of electrospinning technology and hydrogel materials to achieve this objective.^54^ Our approach not only enhances local drug concentrations but also reduces the risk of systemic side effects through controlled release, a benefit seldom reported in previous research.

The innovative aspect of the Fiber‐SIN/Gel‐LidC composite material lies in its unique synergistic release mechanism. Previous studies predominantly focused on the effects of single drugs, whereas this research enhances therapeutic outcomes and reduces side effects through the controlled physical and chemical release of two drugs, producing a synergistic effect. This strategy is particularly crucial for the treatment of DNP, where pain management often requires a combination of drugs to achieve optimal analgesic effects. Additionally, by integrating electrospinning technology with hydrogel systems, this study optimizes drug release dynamics, offering a platform for controlled release, a feature rarely seen in previous research.

The use of electrospinning technology and hydrogel materials in this study provides a new direction for drug delivery systems design. Compared to traditional drug carriers such as microparticles and liposomes, Fiber‐SIN/Gel‐LidC ensures more stable and predictable drug release profiles.^55^ This novel carrier not only improves drug bioavailability but also protects the drugs from premature metabolism or degradation through its unique microenvironment, addressing a gap in previous studies.^19^, ^56^

In summary, this research successfully develops a new composite material based on electrospinning‐hydrogel technology, Fiber‐SIN/Gel‐LidC, for the synergistic release of SIN and Lid to treat DNP. Our findings further validate the mechanism of alleviating neuropathic pain by modulating MMP9 expression, which differs from previous strategies that relied on a single target. This multi‐target intervention strategy demonstrates the potential to manage DNP by comprehensively regulating inflammatory and neuroprotective pathways.

The Fiber‐SIN/Gel‐LidC composite material demonstrates significant clinical potential for treating DNP, yet its path from laboratory research to clinical application is fraught with challenges. Although this study has achieved promising therapeutic results in animal models, providing a scientific basis for further application, these results must be validated in humans. This necessitates large‐scale clinical trials to ensure safety and efficacy. Additionally, commercial production of the composite material requires overcoming technical challenges, including scaling up production, stringent quality control, and ensuring long‐term stability during storage and transport. These are key issues that must be addressed in future research and development.

Despite the encouraging outcomes, several limitations should be acknowledged. First, the present study focused primarily on short‐term drug release behavior and therapeutic efficacy, while long‐term pharmacokinetics and drug stability remain to be explored in future investigations. Although MMP9 was successfully identified through bioinformatics prediction and functional enrichment analysis as a key regulatory target of Fiber‐SIN/Gel‐LidC, we did not conduct direct functional validation (e.g., gene knockdown or pharmacological inhibition). We therefore plan to perform such validation in future studies to clarify the specific mechanisms of MMP9 regulation in vivo. Moreover, while the diabetic rat model used in this study replicates certain aspects of human pathology, physiological and pathological differences remain, which may limit the translatability of the findings. Mechanistically, although we analyzed NF‐κB activation and TIMP expression levels and preliminarily uncovered potential downstream regulatory pathways of MMP9, the causal relationship has not been fully established. Further experiments, such as siRNA knockdown or pharmacological inhibition, are needed to verify MMP9's functional role. Additionally, since NF‐κB and TIMPs may also be regulated by other inflammatory and extracellular matrix‐related factors, future studies should explore the interplay among these molecules to comprehensively elucidate the role of MMP9 in diabetic neuropathic pain. Based on molecular docking analysis, we further speculate that Fiber‐SIN/Gel‐LidC may exert neuroprotective effects by modulating NF‐κB activation or suppressing TIMPs.^57^ Although we observed a reduction in MMP9 expression following treatment, the precise mechanism by which MMP9 influences NF‐κB or TIMP signaling remains unclear and warrants further investigation. In addition, considering the ethical 3R principles and sample sizes reported in prior studies (e.g., PMID: 30838902 and 34163328), we used six animals per group. Although this number does not meet the recommended sample size for power analysis, statistically significant differences were still observed. Despite these limitations, our findings provide important insights into the regulatory role of Fiber‐SIN/Gel‐LidC in MMP9 modulation and lay a foundation for future mechanistic exploration and preclinical validation.

In summary, the Fiber‐SIN/Gel‐LidC composite material, through its unique synergistic drug release mechanism, effectively treats DNP and demonstrates significant neuroprotective effects. These findings not only provide a new therapeutic strategy for DNP but also open new research directions for the treatment of other types of chronic pain. Future research will further explore the clinical potential of this composite material, optimize its drug release properties, and validate its safety and efficacy in preclinical and clinical trials. Furthermore, our experimental results suggest that MMP9 may mediate neuroprotection through the NF‐κB signaling pathway and TIMP‐related matrix preservation mechanisms. This discovery highlights MMP9 as a critical regulatory target and offers new opportunities for DNP treatment. Ongoing studies will continue to explore the functions of MMP9 and its downstream pathways, integrating gene knockdown and pharmacological inhibition strategies to systematically assess its clinical utility in chronic pain management. Additionally, as this study focused on the in vivo pharmacokinetic characteristics of the drug delivery system, substantial prior evidence has demonstrated the key role of the Fiber‐SIN/Lid‐LC composite in promoting sustained drug release and enhancing therapeutic efficacy. This aligns closely with our goal of achieving long‐term local pain relief. To further refine the mechanistic understanding, we plan to utilize patch‐clamp techniques in future studies to directly record Nav1.7 and Nav1.8 sodium channel currents, thereby determining whether Fiber‐SIN/Gel‐LidC exerts its analgesic effects via direct inhibition of these channels. Through these studies, we aim to provide more effective and safer treatment options for patients with diabetes and other chronic conditions.

## AUTHOR CONTRIBUTIONS

Wen Chen, Ji Chen, and Yingqing Lu contributed equally to the conceptualization, experimental design, and manuscript preparation. Wen Chen and Fengrui Yang supervised the study and coordinated in vivo experiments. Ji Chen conducted pharmacological and bioinformatics analyses. Yingqing Lu was responsible for material fabrication and characterization. Yangyuxi Chen assisted in data collection and analysis. Xinxin Liu contributed to histological and behavioral evaluations. Fengrui Yang provided critical revisions and secured funding. All authors reviewed and approved the final manuscript.

## CONFLICT OF INTEREST STATEMENT

The authors declare no conflicts of interest.

## ETHICS STATEMENT

All animal experiments were approved by the Animal Ethics Committee of Hunan University of Medicine General Hospital (No. 202403055). This study was approved by the Clinical Ethics Committee of Hunan University of Medicine General Hospital (No. [2022] 58).

## Supporting information


**Figure S1.** Preparation and characterization of LidC.
(a) Schematic diagram of LidC synthesis. (b) SEM images of LidC stored at 25°C, 4°C, and Lid stored at 4°C. (c) XRD patterns of LidC and Lid. (d) FT‐IR spectra of LidC and Lid. (e, f) UV–visible spectra for determining the content of LidC, (e) shows the absorption spectra at different concentrations, (f) shows the standard curve. (g) In vitro release of LidC microcrystals of different lengths. The data at different time points were analyzed using a two‐way ANOVA. All values are presented as the mean ± SEM (*n* = 3).

**Figure S2.** Preparation and characterization of Fiber‐SIN.
(a) SEM observation of PLGA electrospun fiber morphology, bar = 20 μm. (b) Average diameter of PLGA fibers. (c) Average diameter of PLGA fibers loaded with SIN. The experiment was repeated three times (*n* = 3).

**Figure S3.** Injectable verification of 40% F127.

**Figure S4.** Verification of DNP modeling.
(a, b) Thermal hyperalgesia threshold (a) and mechanical hyperalgesia threshold (b) at different time points after model creation in rats. The data at different time points were analyzed using a two‐way analysis of variance (ANOVA), with six rats per group (*n* = 6), ***p < 0.001.

**Figure S5.** Biocompatibility assessment of Fiber‐SIN/Gel‐LidC. (a, b) Changes in liver function indicators in different treatment groups (ALT: alanine aminotransferase, AST: aspartate aminotransferase, ALP: alkaline phosphatase, TP: total protein, ALB: albumin). (c) Changes in kidney function indicators in different treatment groups (CR: creatinine, BUN: blood urea nitrogen). (d) Changes in white blood cell count in different treatment groups. (e) Changes in platelet count in different treatment groups. (f) Observation of tissue slices in different treatment groups, with the scale marker = 100 μm. The cell experiments were performed three times, with six mice per group (*n* = 6). Multiple group comparisons were conducted using one‐way ANOVA.

**Table S1.** Methods and parameters for producing stable and injectable electrospun nanofibers.
**Table S2.** Methods for producing thermo‐sensitive and injectable hydrogel.
**Table S3.** Detection of modeling indexes in DNP rats.
**Table S4.** The effect of f‐GAS/g‐LibC on active membrane properties of capsaicin‐sensitive DRG neurons.

## Data Availability

All data can be provided as needed.
