# Supplementary figures and images for: Fiber‐Electrospun Hydrogel Therapy for DNP: A synergistic electrospun‐hydrogel composite for alleviating diabetic neuropathic pain via MMP9 regulation and sodium channel inhibition
Source: Bioeng Transl Med. 2025 Jul 28;11(3):e70050. doi: 10.1002/btm2.70050 (PMC13247398; doi:10.1002/btm2.70050)

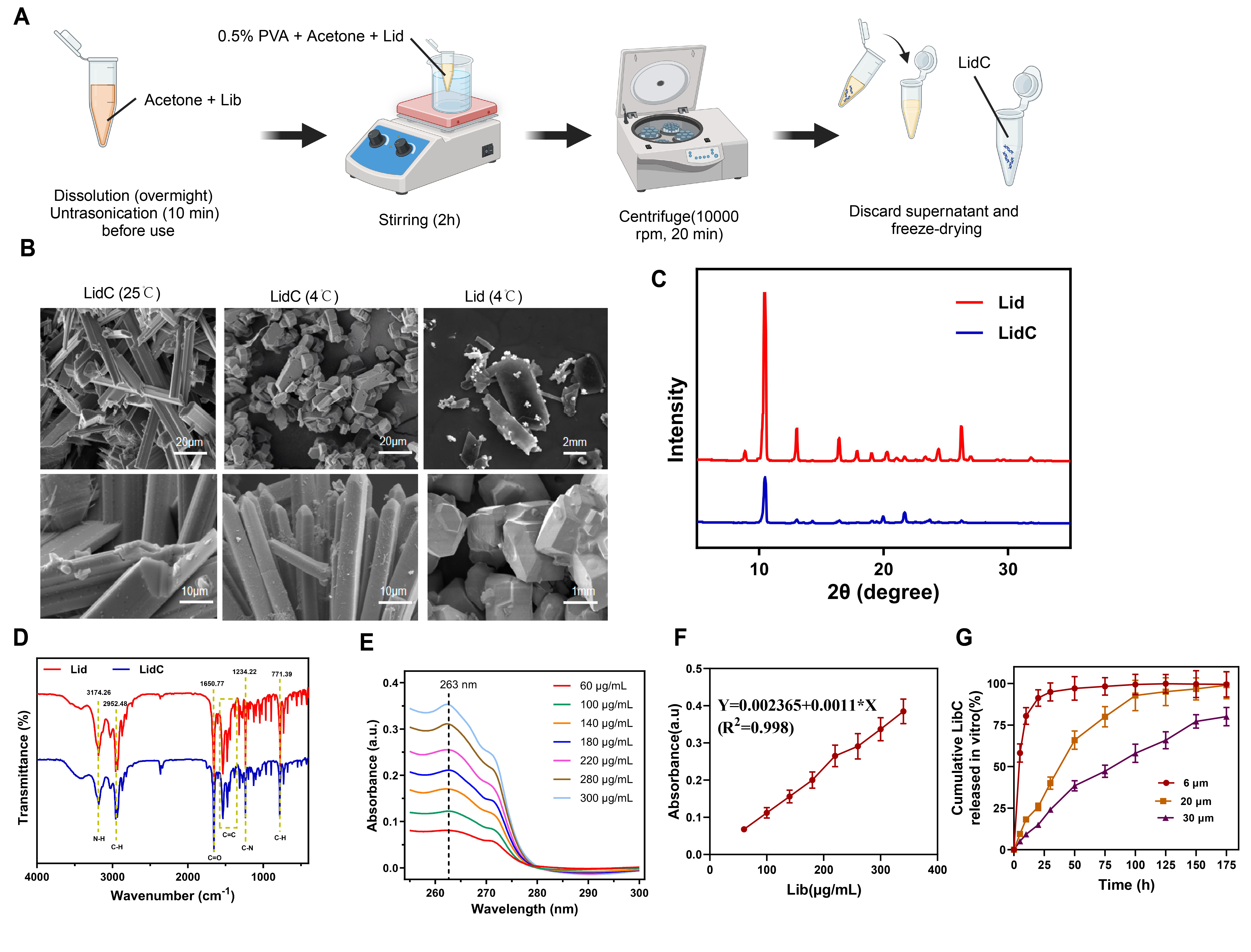

Supplement: Supplementary file 1 — Figure S1. Preparation and characterization of LidC. (a) Schematic diagram of LidC synthesis. (b) SEM images of LidC stored at 25°C, 4°C, and Lid stored at 4°C. (c) XRD patterns of LidC and Lid. (d) FT‐IR spectra of LidC and Lid. (e, f) UV–visible spectra for determining the content of LidC, (e) shows the absorption spectra at different concentrations, (f) shows the standard curve. (g) In vitro release of LidC microcrystals of different lengths. The data at different time points were analyzed using a two‐way ANOVA. All values are presented as the mean ± SEM (n = 3). [file BTM2-11-e70050-s005.jpg]

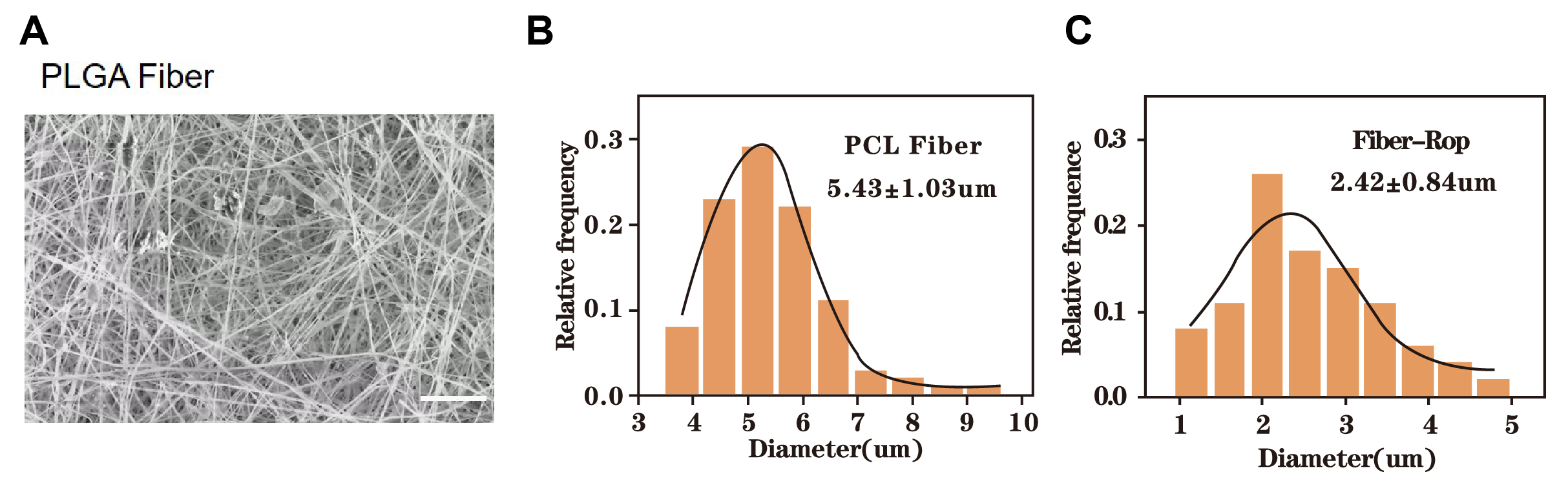

Supplement: Supplementary file 2 — Figure S2. Preparation and characterization of Fiber‐SIN. (a) SEM observation of PLGA electrospun fiber morphology, bar = 20 μm. (b) Average diameter of PLGA fibers. (c) Average diameter of PLGA fibers loaded with SIN. The experiment was repeated three times (n = 3). [file BTM2-11-e70050-s004.jpg]

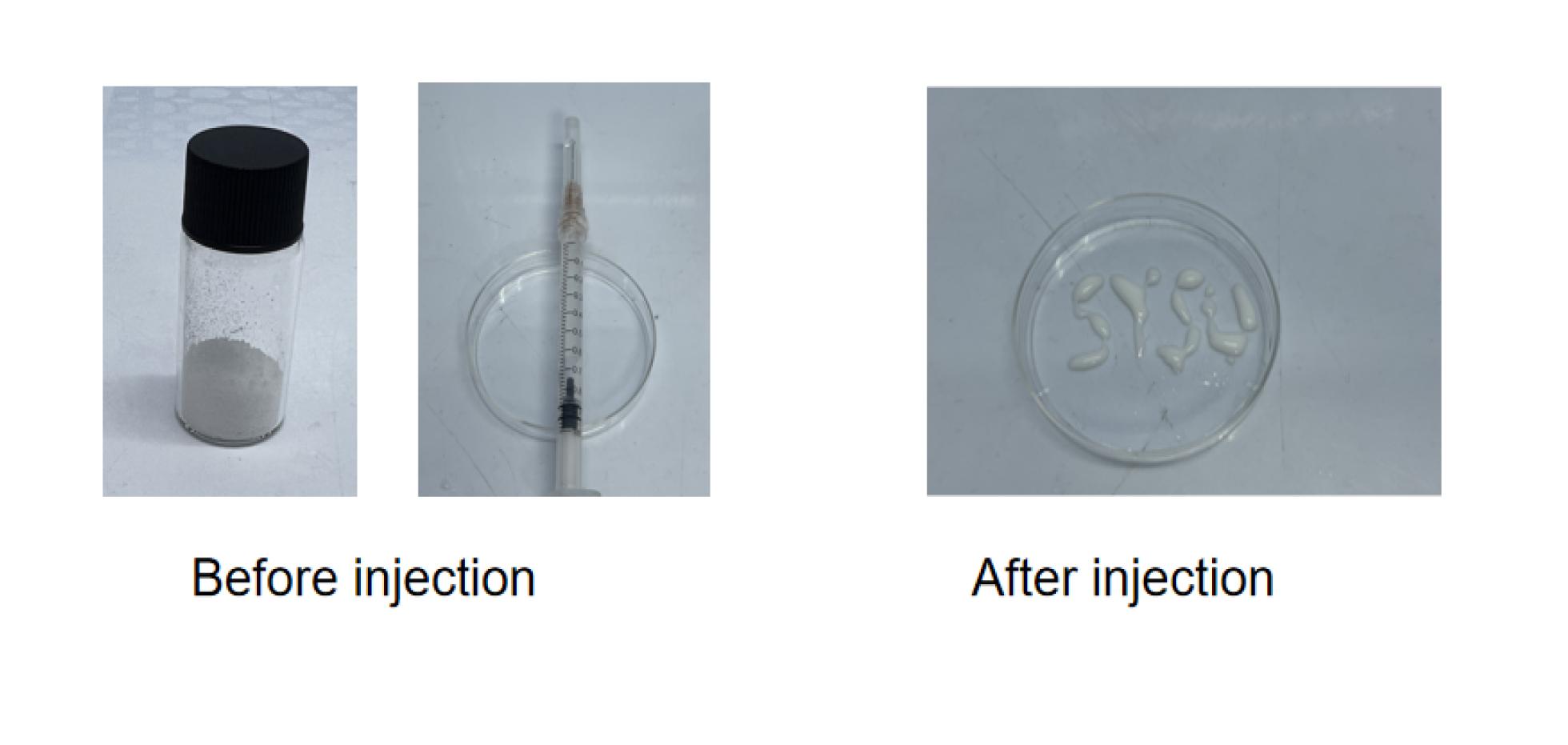

Supplement: Supplementary file 3 — Figure S3. Injectable verification of 40% F127. [file BTM2-11-e70050-s006.jpg]

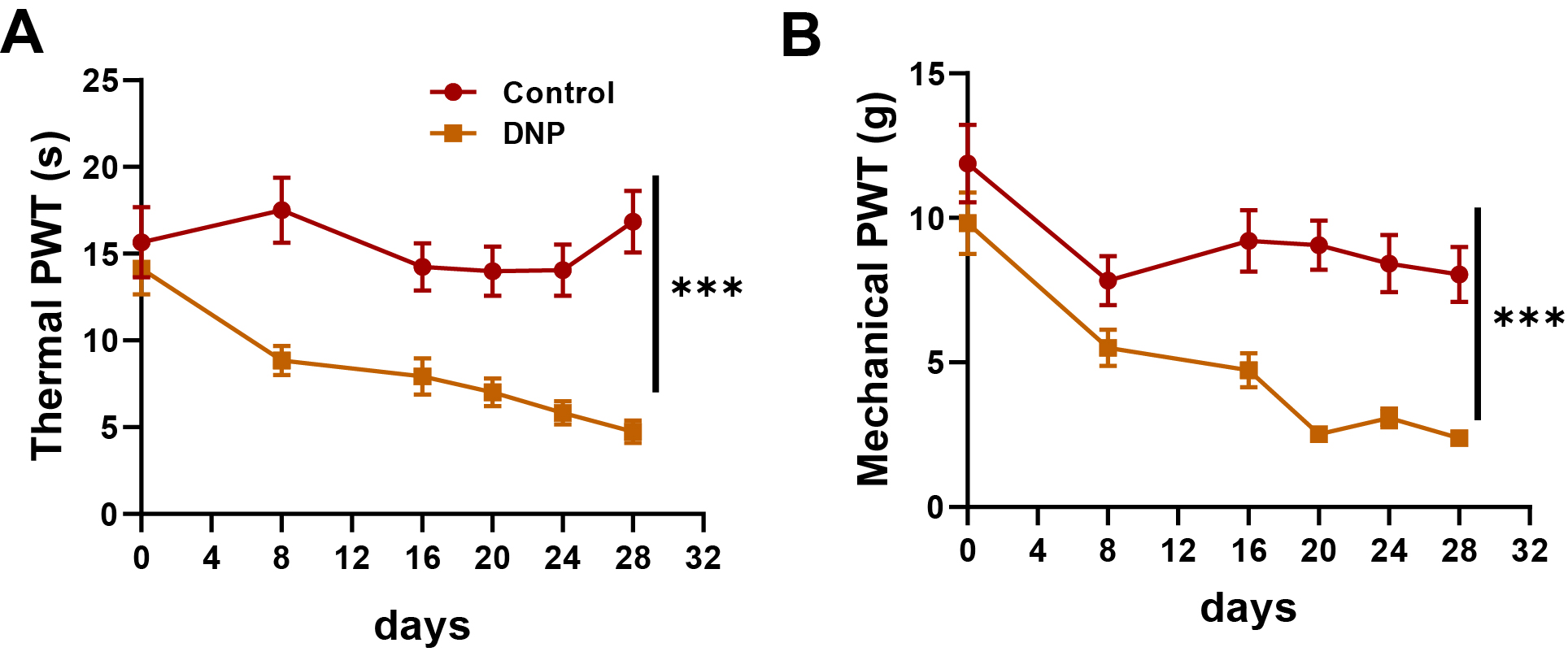

Supplement: Supplementary file 4 — Figure S4. Verification of DNP modeling. (a, b) Thermal hyperalgesia threshold (a) and mechanical hyperalgesia threshold (b) at different time points after model creation in rats. The data at different time points were analyzed using a two‐way analysis of variance (ANOVA), with six rats per group (n = 6), ***p < 0.001. [file BTM2-11-e70050-s003.jpg]

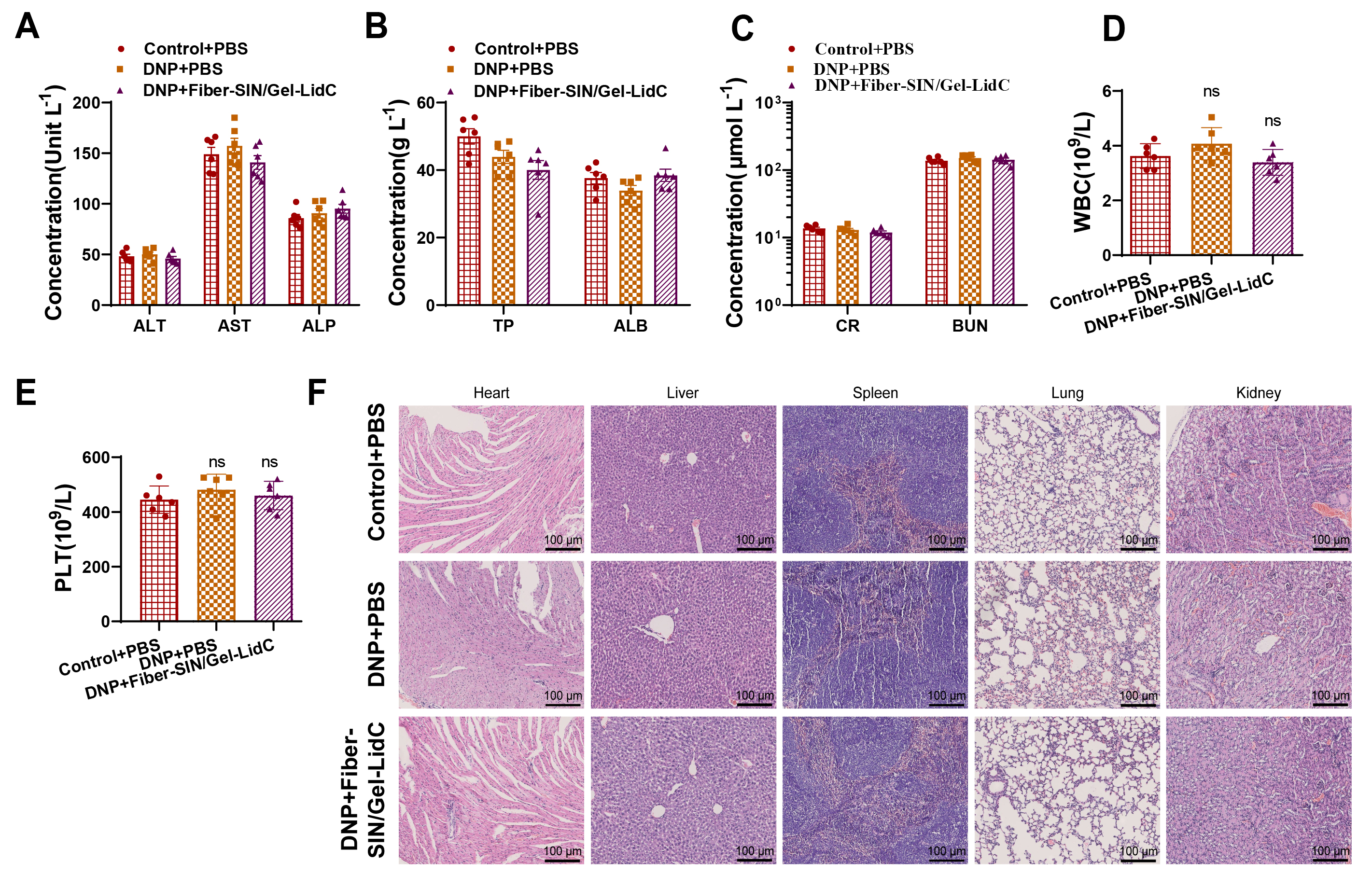

Supplement: Supplementary file 5 — Figure S5. Biocompatibility assessment of Fiber‐SIN/Gel‐LidC. (a, b) Changes in liver function indicators in different treatment groups (ALT: alanine aminotransferase, AST: aspartate aminotransferase, ALP: alkaline phosphatase, TP: total protein, ALB: albumin). (c) Changes in kidney function indicators in different treatment groups (CR: creatinine, BUN: blood urea nitrogen). (d) Changes in white blood cell count in different treatment groups. (e) Changes in platelet count in different treatment groups. (f) Observation of tissue slices in different treatment groups, with the scale marker = 100 μm. The cell experiments were performed three times, with six mice per group (n = 6). Multiple group comparisons were conducted using one‐way ANOVA. [file BTM2-11-e70050-s001.jpg]
