# Supplementary material for: Fiber‐Electrospun Hydrogel Therapy for DNP: A synergistic electrospun‐hydrogel composite for alleviating diabetic neuropathic pain via MMP9 regulation and sodium channel inhibition
Source: Bioeng Transl Med. 2025 Jul 28;11(3):e70050. doi: 10.1002/btm2.70050 (PMC13247398; doi:10.1002/btm2.70050)
Supplement: Supplementary file 6 — Table S1. Methods and parameters for producing stable and injectable electrospun nanofibers. Table S2. Methods for producing thermo‐sensitive and injectable hydrogel. Table S3. Detection of modeling indexes in DNP rats. Table S4. The effect of f‐GAS/g‐LibC on active membrane properties of capsaicin‐sensitive DRG neurons. [file BTM2-11-e70050-s002.docx]

**Table S1. Methods and parameters for producing stable and injectable electrospun nanofibers**

| **Materials** | **Solvent(v/v)** | **Parameters** | **Stable Fiber** | **Injectable** |
| --- | --- | --- | --- | --- |
| 0%PLGA: GAS(3: 1) | DCM/DMF 70/30 | 0.8mL/h, 20cm, 15kV | No | No |
| 10%PLGA: GAS(3: 1) | DCM/MeOH 70/30 | 0.8mL/h, 20cm, 15kV | No | No |
| 10%PLGA: GAS(2: 1) | DCM/DMF 70/30 | 1.5mL/h, 20cm, 20kV | Yes | No |
| 10%PLGA: GAS(2: 1) | DCM/MeOH 70/30 | 1.5mL/h, 20cm, 20kV | Yes | No |
| 10%PLGA: GAS(1: 1) | DCM/DMF 70/30 | 0.8mL/h, 20cm, 20kV | Yes | No |
| 10%PLGA: GAS(1: 1) | DCM/MeOH 70/30 | 0.8mL/h, 20cm, 20kV | No | No |
| 15%PLGA: GAS(1: 1) | DCM/DMF 70/30 | 2.4mL/h, 20cm, 18kV | No | No |
| 15%PLGA: GAS(1: 1) | DCM/MeOH 70/30 | 2.4mL/h, 20cm, 18kV | No | No |
| 20%PLGA: GAS(1: 1) | DCM/DMF 70/30 | 1.2mL/h, 20cm, 20kV | No | No |
| 20%PLGA: GAS(1: 1) | DCM/MeOH 70/30 | 1.2mL/h, 20cm, 20kV | Yes | No |
| 20%PLGA: GAS(1: 2) | DCM/DMF 70/30 | 0.8mL/h, 20cm, 20kV | Yes | Yes |
| 20%PLGA: GAS(1: 2) | DCM/MeOH 70/30 | 0.8mL/h, 20cm, 20kV | Yes | No |

**Table S2. Methods for producing thermo-sensitive and injectable hydrogel**

| **Hydrogel Architecture (Wt%)** | **Time to Form Hydrogel at Room Temperature (24 ± 0.5 ℃)** | **Time to Form Hydrogel at 37 ℃** |
| --- | --- | --- |
| 20% F127 | no | no |
| 25% F127 | no | 5 min |
| 30% F127 | 10 min | 20 s |
| 35% F127 | 4 min | 10 s |
| 40% F127 | 2 min | immediate |

**Table S3. Detection of modeling indexes in DNP rats**

| **Groups** | **Control** | **DNP** |
| --- | --- | --- |
| Serum Glucose (mg/dI) | 90.53±1.56 | 421.67± 3.61* |
| Serum Insulin (μIU/ml) | 15.81±0.23 | 6.67±0.18* |
| Body Weight (gm) | 281.00±4.08 | 221.33 ±3.76* |

Note: *p<0.05, represents significant difference between Control and DNP.

**Table S4. The effect of f-GAS/g-LibC on active membrane properties of capsaicin-sensitive DRG neurons.**

|  | **Apamplitude**  **(mv)** | **Aphalf-**  **width(ms)** | **Apmax-rise**  **Slope(mv/ms)** | **Apmax-decay Slope(mv/ms)** | **Apthreshold(mv)** |
| --- | --- | --- | --- | --- | --- |
| Control+PBS | 98.57±2.94 | 2.77±0.21 | 96.18±5.37 | -87.45±5.14 | -26.67±2.29 |
| DNP+PBS | 115.39±3.29 | 2.01±0.18 | 124.23±11.55* | -72.32±8.43 | -37.39±2.68 |
| DNP+f-GAS/g-LibC | 92.32±2.85# | 2.36±0.20 | 98.77±5.21# | -98.52±6.54 | -22.56±2.32# |

Note: * p<0.05, represents significant difference between Control and DNP; # p<0.05, represents significant difference between DNP+f-GAS/g-LibC and DNP.
